# Native Mass Spectrometry Imaging of Proteins and Protein Complexes by Nano-DESI

**DOI:** 10.1021/acs.analchem.0c05277

**Published:** 2021-03-04

**Authors:** Oliver J. Hale, Helen J. Cooper

**Affiliations:** School of Biosciences, University of Birmingham, Edgbaston B15 2TT, U.K.

## Abstract

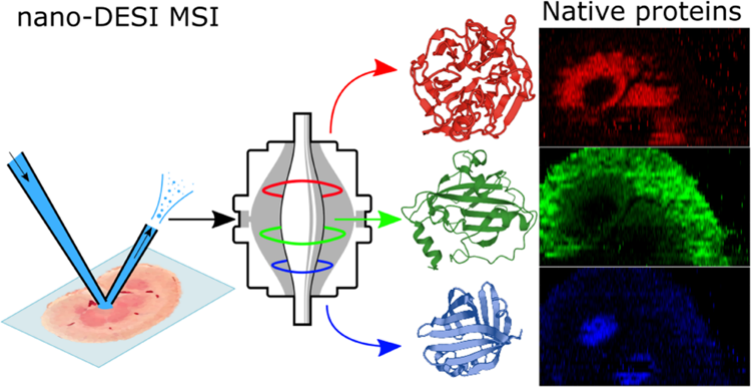

Previously, we have demonstrated native mass spectrometry imaging (native MSI) in which the spatial distribution of proteins maintained in their native-like, folded conformations was determined using liquid extraction surface analysis (LESA). While providing an excellent testbed for proof of principle, the spatial resolution of LESA is currently limited for imaging primarily by the physical size of the sampling pipette tip. Here, we report the adoption of nanospray-desorption electrospray ionization (nano-DESI) for native MSI, delivering substantial improvements in resolution versus native LESA MSI. In addition, native nano-DESI may be used for location-targeted top–down proteomics analysis directly from tissue. Proteins, including a homodimeric complex not previously detected by native MSI, were identified through a combination of collisional activation, high-resolution MS and proton transfer charge reduction.

## Introduction

Native mass spectrometry of proteins is a fast-growing field of mass spectrometry in which the intra- and intermolecular noncovalent interactions present in solution are maintained in the gas phase.^1^ Proteins are subjected to electrospray ionization mass spectrometry from solutions designed to mimic physiological conditions. As noncovalent interactions are retained in the gas phase, it is possible to obtain protein structural information from native mass spectrometry. For example, the stoichiometry of a protein assembly can be determined through mass measurement and tandem mass spectrometry analysis.^2^ For single polypeptide chains, the charge state distribution is indicative of the folded protein (the more folded the protein, the lower the charge state). Often, native mass spectrometry is coupled with ion mobility spectrometry for the purposes of measuring collision cross sections.^3^ Structural information is much sought-after because of its link to protein function.

Mass spectrometry imaging (MSI) allows simultaneous detection of biological molecules in an untargeted approach, a useful trait for discovery biology applications.^4^ The first MSI publication for protein imaging used matrix-assisted laser desorption/ionization mass spectrometry (MALDI MS), as have many later studies.^4−6^ Proteins have also been imaged by first conducting an on-tissue proteolytic digest, followed by MALDI MS analysis.^7,8^ In that approach, peptide markers of the protein are analyzed rather than the intact protein. More recently, ambient ionization mass spectrometry imaging methods, such as desorption electrospray ionization (DESI) and nanospray-desorption electrospray ionization (nano-DESI), have been developed.^9−12^ To date, these methods have made use of denaturing solvents which result in loss of information on protein complex stoichiometry and three-dimensional structure. This information is valuable for understanding interactions with other proteins and small molecules, which are often critical to protein function. As described above, native mass spectrometry can retain that structural information in the gas phase and it has become an established tool for investigating protein structure and binding, usually from purified recombinant proteins.^1,13,14^ Consequently, we have endeavored to combine MSI and native MS for intact protein imaging.

Our existing studies on native MSI have all used liquid extraction surface analysis (LESA) as the sampling and ionization platform.^15,16^ LESA is automatable and sensitive, but is limited in its achievable spatial resolution by the size of the pipette tip used for sampling, and the step increment of the motors on the robot. Typically, LESA results in a sampling location area with a diameter of 600 μm, and pixel size of 1 × 1 mm^2^. Substantial improvement of spatial resolution is therefore a key focus to make native MSI a more powerful tool for biology, particularly as MSI of small molecules is now routinely achievable at cellular resolutions.^17,18^ Alternative ambient ionization techniques were considered for adaption for native MSI: desorption electrospray ionization (DESI) has been successfully applied to protein imaging under denaturing conditions with the assistance of ion mobility separation,^9,10^ and is capable of native analysis of purified proteins applied to a glass slide,^19^ but requires harsh desolvation conditions. Nanospray-desorption electrospray ionization (nano-DESI),^20,21^ which employs direct contact of a flowing solvent bridge with the sample to dissolve molecules prior to ionization, has also been applied to denatured protein MSI.^11,12^ Nano-DESI has been used to achieve lateral spatial resolutions of around 10 μm when analyzing abundant lipid and metabolite species,^22^ and around 200 μm for denatured proteins.^11,12^ Pixel sizes of 5 μm have been achieved with microscopy-assisted image fusion, where lower resolution ion images are merged with a high-resolution optical image.^12^

Here, we present the first report of native nano-DESI MSI enabling significant improvements in spatial resolution over LESA MSI. Native nano-DESI makes use of a nondenaturing ammonium acetate-based aqueous solvent for analyzing the spatial distribution of intact proteins and complexes. (Duncan et al. have previously demonstrated nano-DESI MSI using pure water for imaging of metabolites in kidney tissue sections.^23^) Identification of the endogenous proteins is also crucial for the viability of the technique. We demonstrate top–down proteomics of native proteins directly from rat kidney tissue using nano-DESI, including identification of the noncovalent dimer of S100-A6, adipocyte lipid-binding protein (ALBP), and phosphatidylethanolamine-binding protein (PEBP1), none of which have previously been identified by native LESA.

## Materials and Methods

MS-grade water, acetonitrile (ACN), methanol (MeOH), and formic acid (FA) were purchased from Fisher Scientific (Loughborough, U.K.). High-performance liquid chromatography (HPLC)-grade ammonium acetate was bought from J.T. Baker (Deventer, The Netherlands). All protein standards and C_8_E_4_ were bought from Sigma-Aldrich (Gillingham, U.K.). Solutions of hen egg white lysozyme (HEWL, 160 μM), equine skeletal muscle myoglobin (670 μM), and ovalbumin (420 μM) were made up in aqueous ammonium acetate (200 mM) to mimic the physiological electrolyte concentration.^24^ In the initial investigation, samples were deposited as 1 μL droplets onto a glass slide and allowed to dry at room temperature for 30 min. The dried droplets were typically 5–8 mm in diameter for 1 μL deposits. The dried droplets were then sampled by nano-DESI with 200 mM ammonium acetate. Subsequent limit of detection (LOD) experiments were performed using 0.2 μL dried droplets (typically <2 mm diameter).

The solvent system used for producing ion images of rat kidney was ammonium acetate (200 mM) + 0.125% C_8_E_4_ detergent. Detergent-based solvent systems are common in native MS studies of membrane proteins for stabilizing gas-phase structure, and this solvent system was previously used in native LESA experiments for analyzing intact protein assemblies.^25−27^ Additional experiments featuring organic solvents used solutions of ACN/0.1% FA (80:20, 65:35, 50:50 v/v) and MeOH/0.1% FA (80:20 v/v). Nitrogen (>99.995%) and helium (>99.996%) gases used on the mass spectrometer were obtained from BOC (Guildford, U.K.).

### Animal Tissue

Kidney tissue from a vehicle-dosed (0.5% hydroxypropyl methylcellulose (HPMC) and 0.1% Tween 80 in water) adult male Hans Wistar rat was the kind gift of Dr. Richard Goodwin (Astra Zeneca). The animal was euthanized 2 h post dose. Dissection was performed by trained Astra Zeneca staff (project licence PP77366793, procedure number 3). Kidneys were snap-frozen in isopropanol over dry ice. All tissue was stored at −80 °C and sectioned at −22 °C to a thickness of 10 μm with a CM1810 Cryostat (Leica Microsystems, Wetzlar, Germany) and thaw mounted onto glass microscope slides. Sections were stored at −80 °C until use. Three tissue sections from various depths through the organ (∼20, ∼40, and ∼60%) were subjected to replicate analysis by native nano-DESI MSI. Tissue sections were not subjected to any washing protocol prior to analysis to avoid protein unfolding or complex dissociation due to additional preparation.

### Nano-DESI Ion Source

Nano-DESI was performed using a custom-built ion source, based on the two-dimensional (2D) XY-stage of a FlowProbe (Prosolia, Inc., Indianapolis, IN) and taking inspiration from the nano-DESI source of Laskin and co-workers.^28^ The *Z*-axis was a translating platform positioned by a micrometer screw. Solvent was supplied by a syringe pump (500 μL, Hamilton, Reno, NV) at a flow rate of 1.9 μL/min through fused silica capillaries (OD = 280 μm, ID = 75 μm before modification). The fused silica was flame pulled to produce a taper and then cut approximately 2 mm from the tip to reveal the orifice (see Figure S1a). The end of the capillary at the mass spectrometer inlet was not flame pulled, but had the capillary coating removed. The primary and sampling capillaries were positioned at an angle of approximately 90° to one another to form an opening over which a liquid junction could form. Initially, the sampling capillary was approximately 10 cm long (used for sampling protein standards), but this was shortened to approximately 2.5 cm for tissue imaging. The shorter capillaries have proven less liable to blocking by particulates and bubbles. The sampling capillary was positioned ∼0.5 mm inside the ion transfer tube of the mass spectrometer, which enabled aspiration of the solvent by the inlet vacuum. Microscope cameras (Dino-Lite, Torrance, CA) were mounted to aid positioning of the capillaries. A potential between 800 and 1600 V (tuned for signal stability and intensity) was applied to the solvent by a high voltage cable from the mass spectrometer connected to the solvent syringe (see Figure S1b,c). The 2D stage was controlled by Arduino Uno v3 microcontrollers (Arduino, Turin, Italy), EasyDriver stepper motor drivers (v4.4, http://www.schmalzhaus.com) and custom scripts in the Arduino software (v1.8.12) (see Figure S2). For protein standards, the nano-DESI liquid junction was formed on the glass slide surface. The 2D stage was moved in 100 μm increments toward the edge of a dried droplet until protein ions were detected, after which movement was stopped and data were acquired (static nano-DESI). For tissue imaging, the nano-DESI probe was brought into contact with a test tissue section and the solvent bridge formed. The stage then moved to an adjacent tissue section for imaging. The motorized stage was set to move at a rate of 20 μm/s laterally, and to step 200 μm between line scans. Line scans were acquired left to right as seen in images in this article. The kidney images shown here required approximately 7 h to acquire.

### Mass Spectrometry

The nano-DESI source was mounted to an Orbitrap Eclipse (Thermo Fisher, San Jose, CA) equipped with the HMR^n^ option, allowing *m*/*z* analysis and selection up to *m*/*z* 8000. The Orbitrap Eclipse was operated in positive-ion mode and intact protein mode with the ion routing multipole pressure set to 8 mTorr. Tuning and calibration were performed using FlexMix (Thermo Fisher). The electrodynamic ion funnel RF was set to 140%, with source-induced dissociation set to 85 V for kidney, optimized for removal of background ions and improved protein ion signal intensity. Data were acquired in the orbitrap analyzer at a resolution setting of 15 000 or 60 000 (defined at *m*/*z* 200) for protein standards and 7500 for tissue analysis. The Orbitrap Eclipse mass spectrometer is optimized for intact protein analysis under native conditions.^29^ Low-resolution mass analysis is the result of collection of a shorter orbitrap transient, e.g., the transient length for a resolution of 7500 is only 16 ms. The short transient is beneficial to the signal intensity of larger proteins. Longer transients increase the probability of signal decay by the phenomena described by Makarov and Denisov.^30^ An *m*/*z* range of 2000–5000 for kidney tissue was used to exclude high intensity singly charged ions, such as lipid dimers, which were observed to be multiple orders of magnitude more intense than protein ion signals. Decreasing the lower *m*/*z* limit to include lipid dimer signals (∼*m*/*z* 1500) resulted in rapid automatic gain control (AGC) triggering and an absence of protein signals. Injection time for full scan spectra was set to 500 ms, and the AGC set to 1250%, which generally resulted in between 2 and 5 spectra/s.

Tandem mass spectrometry (MS^n^) experiments used the ion trap for *m*/*z* selection. Ions were dissociated by higher-energy collisional dissociation (HCD) or collision-induced dissociation (CID). Normalized collision energy (NCE) values are specified alongside each MS^n^ spectrum for each protein identified in this study. For product-ion detection, an Orbitrap resolution between 50 000 and 500 000, or ion trap detection with the “Normal” scan rate was used. MS^n^ experiments were performed on an adjacent section of kidney to the one imaged. MS^n^ spectra were manually interrogated to build a zero-charge peak list, which was imported into ProSight 4.1 (Thermo). Searches were performed with the proteome of *Rattus norvegicus* (Uniprot Proteome: UP000002494, downloaded June 2020). Precursor monoisotopic mass tolerance was set to 1 kDa to allow for hits including small bound ligands, fragment ion tolerance of 20 ppm and a minimum fragment match of 1. Assignment of MS^n^ signals was checked manually using MS-Product (ProteinProspector, v5.24.0, http://prospector.ucsf.edu/prospector/mshome.htm, UCSF) to predict fragment *m*/*z*.

For proton transfer charge reduction (PTCR) experiments, the reagent anion was perfluoroperhydrophenanthrene. PTCR charge reduces protein cations by an ion/ion reaction in the high-pressure cell of the ion trap.^31^ PTCR was performed with a reaction time of 1–10 ms (analyte-dependent), a max. fill time of 200 ms and an AGC target of 2 × 10^5^ for reagent anions. The Orbitrap was set to a resolution of 7500.

### Ion Image Generation

Data were acquired as single line scans, examples of which can be found in Figure S3. All line scans per image were converted from Thermo raw to imzML by FireFly (v3.2.0.23, Prosolia, Inc.) using fixed time bins to box sum multiple scans to account for variability in Orbitrap injection time due to AGC. The imzML files were processed with MSiReader^32^ (v1.02, North Carolina State University, Raleigh, NC). Kidney ion images were produced with a pixel size of 200 × 200 μm^2^. TIC normalization and linear interpolation were applied. All images represent a window across the protein signal apex, specified as *m*/*z* ±0.1.

## Results and Discussion

### Native Nano-DESI MSI

We pursued the application of native nano-DESI to imaging thin tissue sections after initial investigation of protein standards (hen egg white lysozyme (HEWL), holo-myoglobin, and ovalbumin) using the nano-DESI probe (see the Native Nano-DESI of Protein Standards section in the Supporting Information and Figure S4). The LOD for the protein standards dried onto glass slides was determined to be ∼0.4 pmol (see Figures S5 and S6).

The nondenaturing solvent system used here for native nano-DESI is known to allow folded proteins and intact protein complexes to retain their structure through ionization to mass analysis.^1,33^ Denaturing solvent systems that have previously been used for nano-DESI protein imaging^11,12^ disrupt the weak interactions of protein higher-order structure and are likely incompatible with the goal of determination of the spatial distribution of intact protein complexes and folded monomeric proteins. The use of ammonium acetate-buffered solvent systems in the 10–200 mM range is common in native MS to maintain a physiological pH.^2^ Detergents are also used to transfer membrane proteins from solution into the gas phase without disrupting structure.^34^ Although signal suppression is expected because of the solvent system, mitigation through the application of source collision energy and optimized ion optics to remove background ions is effective and does not induce unwanted dissociation of protein complexes.

**Figure 1:**
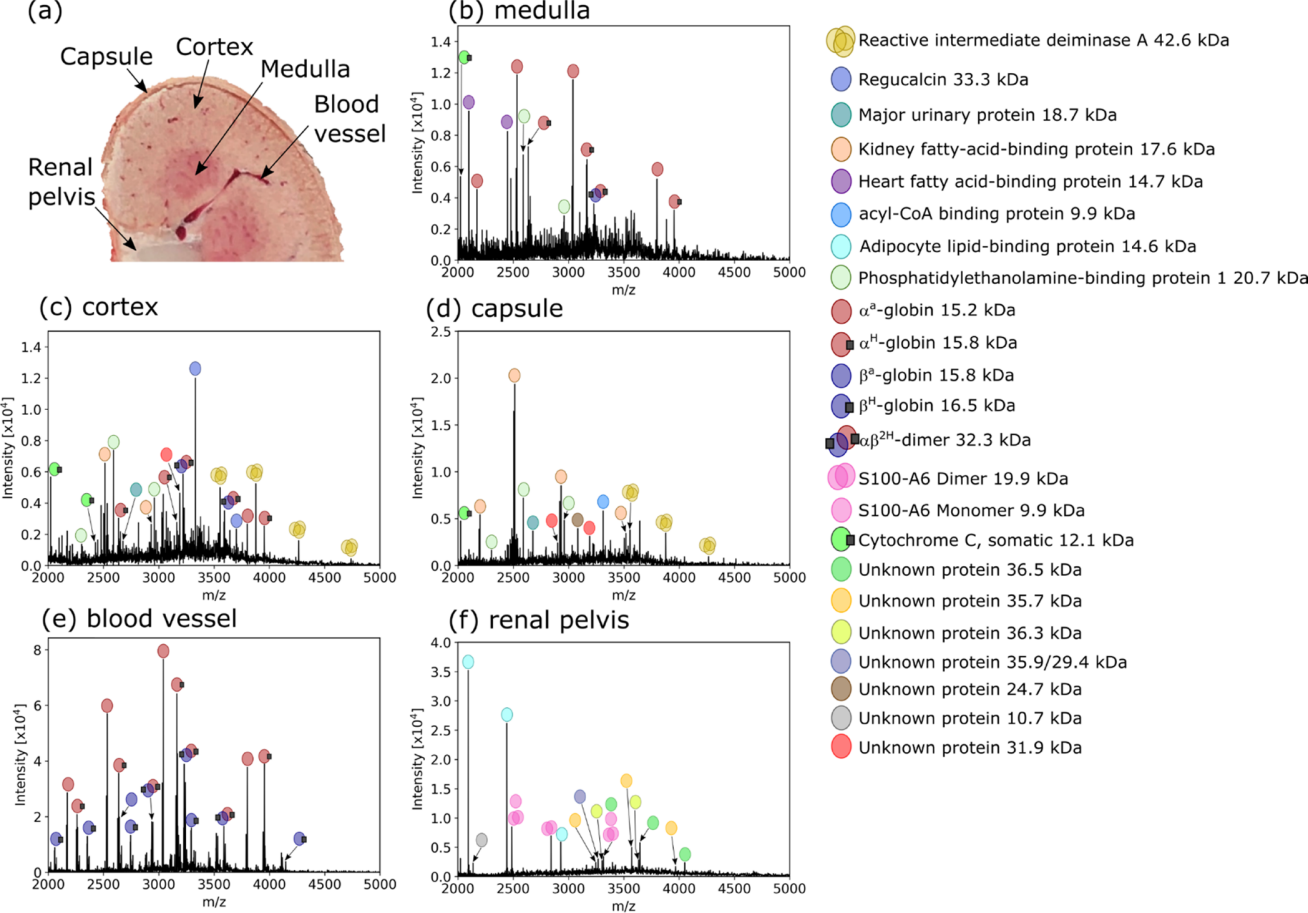
(a) Photograph of the rat kidney during sectioning with labeled regions corresponding to the following mass spectra. (b–f) Native mass spectra from distinct regions of the kidney. Some proteins exhibited strong signals in one region, e.g., H-FABP in the medulla (b), regucalcin in the cortex (c), and adipocyte lipid-binding protein in the renal pelvis (f). Spectra are the result of averaging multiple orbitrap scans ((b): 48, ∼0.096 mm^2^ of tissue sampled, (c): 148, ∼0.3 mm^2^, (d): 18, ∼0.036 mm^2^, (e): 22, ∼0.044 mm^2^, (f): 188, ∼0.376 mm^2^) from raster line scans acquired at 20 μm/s in the respective region of tissue to improve the signal-to-noise ratio.

We have previously investigated rat kidney tissue with native LESA MSI, making for useful comparative data in the form of low-resolution images and protein identifications by an in situ top–down approach.^26^ Representative native nano-DESI mass spectra for specific regions in the kidney are shown in Figure 1. Examples of mass spectra composed of a single scan are available in Figure S7. The proteins labeled were reliably detected in adjacent tissue sections, enabling further MS^n^ analysis to be performed. The medulla tissue features heart fatty acid binding protein (H-FABP), and regucalcin is found only in the cortex tissue spectrum, as was shown with LESA. For nano-DESI, the renal pelvis yielded many signals not observed previously with LESA, possibly because the section used for nano-DESI featured adipose tissue in this region. For example, adipocyte lipid-binding protein (ALBP) was detected with native nano-DESI and is known to be a lipid transport protein found in adipocytes.^35^ Some larger proteins (with MWs ∼36 kDa) with similar distributions to ALBP remain unidentified due to their low relative abundance, but it was possible to determine an average mass for these proteins using PTCR (see below). Other newly identified proteins include the S100-A6 dimer (renal pelvis and medulla) and phosphatidylethanolamine-binding protein 1 (PEBP1; capsule, cortex, and medulla). These identifications were achieved by implementing native nano-DESI MS^n^ (see below).

The increased number of proteins identified by native nano-DESI as compared with native LESA might suggest that native nano-DESI offers greater sensitivity; however, the overall signal intensity is 1–2 orders of magnitude lower on a scan-to-scan basis for native nano-DESI (depending on the protein, see Figure S8 and associated discussion, Supporting Information). We hypothesize that differences in the detected proteins arise because native nano-DESI is a raster technique, which samples all tissue along the line scan, whereas LESA samples from discrete points and with unsampled tissue remaining between points. Importantly, the LESA sampling area (∼0.28 mm^2^) does not equate to pixel size (1 mm^2^) due to software and stepper motor limitations leading to undersampling of the tissue by LESA. Nano-DESI is therefore better positioned to map proteins with limited distributions.

For comparison, we performed nano-DESI analyses of rat kidney with a range of organic solvents (80% ACN, 65% ACN, 50% ACN, and 80% MeOH, each containing 0.1% formic acid). Nano-DESI mass spectra obtained from the cortex, using identical instrument parameters to those used above, are shown in Figure S9. With 80% ACN, only lipid signals were detected. With 65% ACN, a solvent system previously used in nano-DESI protein imaging,^11,12,36,37^ the spectra were dominated by apo-α-globin and apo-β-globin peaks. These peaks were also detected at 50% ACN, together with peaks corresponding to monomers and dimers of RidA. With 80% MeOH, again, peaks corresponding to apo-α-globin and apo-β-globin peaks dominated. As mentioned above, use of the native solvent system requires application of source collision energy to remove background ions, and this is not necessarily the case for the organic solvent systems. We therefore repeated the analyses with organic solvents at a source collision energy of 0 V and the spectra obtained from the cortex are shown in Figure S10. For 80% ACN, 65% ACN, and 80% MeOH, the spectra are dominated by lipid signals, with peaks corresponding to apo-α-globin and apo-β-globin also detected. Interestingly, the RidA trimer was detected in high charge states (13+ to 19+) with 50% ACN. No αβ^2H^ hemoglobin dimers were detected. The experiments with 50% ACN at both source collision energies were repeated for the renal pelvis, see Figure S11. At source collision energy = 0 V, the S100-A6 monomer was detected in a range of charge states. At source collision energy = 85 V, the S100-A6 monomer and cytochrome C were detected. Overall, the use of organic solvents resulted in the detection of fewer proteins than the ammonium acetate-based solvent and unfolding of protein complexes. S100-A6 dimers, αβ^2H^ hemoglobin dimers, and heme-bound α- and β-globins were not detected; RidA trimers were detected when the solvent was 50% ACN; however, the high charge states and facile dissociation of these species suggest some degree of structural disruption under these conditions.

**Figure 2:**
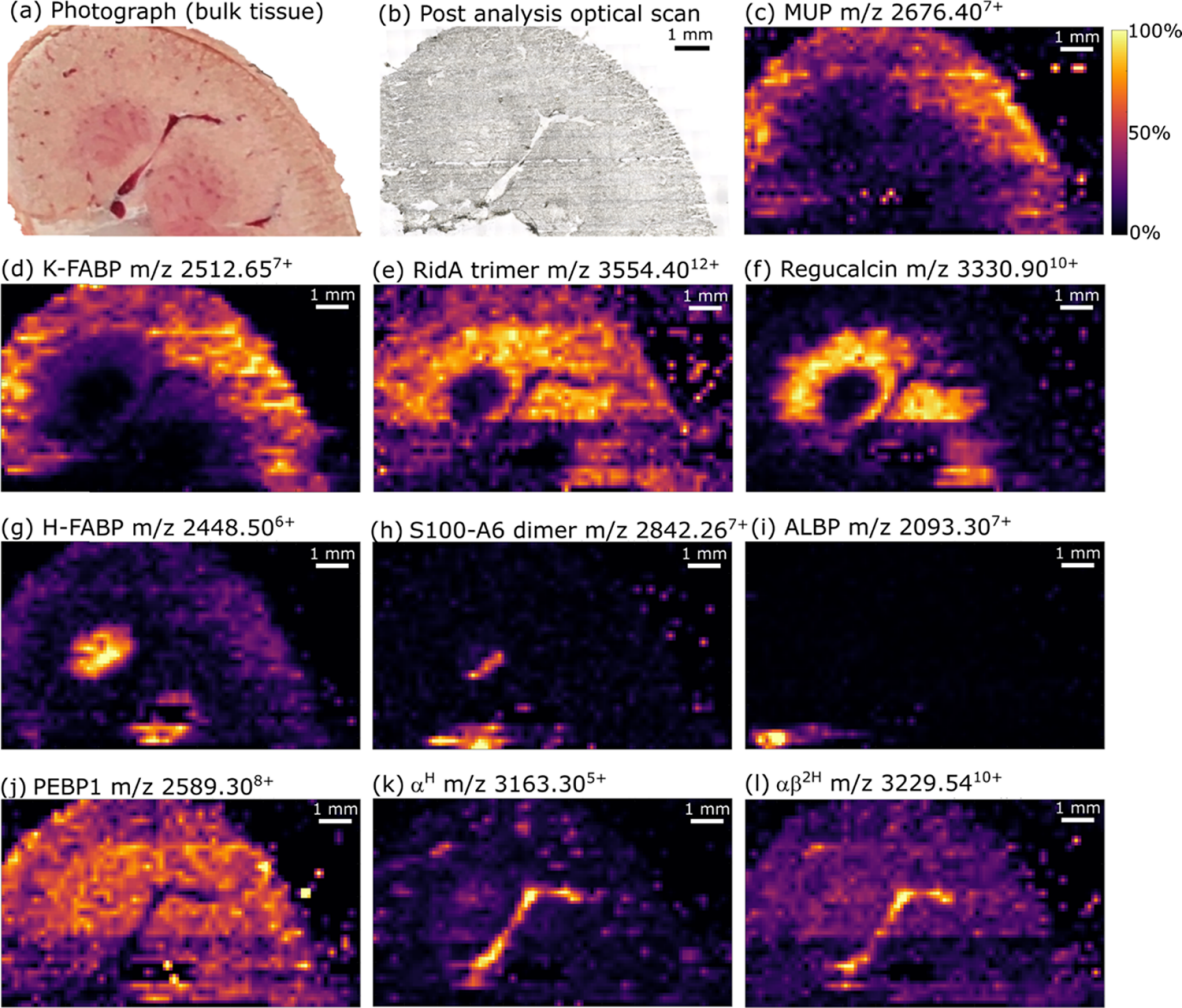
Native nano-DESI mass spectrometry imaging: (a) photograph of the kidney during the production of the section for analysis. (b) Optical image of the kidney section after native nano-DESI analysis. Ion images show the distribution of intact proteins throughout the tissue section; (c) MUP (outer cortex), (d) K-FABP (outer cortex), (e) RidA trimer (cortex), (f) regucalcin (inner cortex), (g) H-FABP (medulla), (h) S100-A6 dimer (medulla, renal pelvis) (i) ALBP (renal pelvis adipose tissue), (j) PEBP1 (bulk tissue), (k) α^H^ (vasculature), and (l) αβ^2H^ (vasculature). Images showing additional charge states are found in Figures S13–S18, S20, S22, S24, and S25.

Ion images obtained from a rat kidney section are shown in Figure 2. Ion images produced for kidney sections in replicate experiments are available in Figure S12. The analyzed section (see photograph of the kidney at sectioning in Figure 2a) contained one prominent blood vessel running from the renal pelvis, up the cortical column (between two renal pyramids of medulla tissue) and into the cortex. Smaller blood vessels are prominent in the cortex tissue. An optical image of the tissue section post-analysis is shown in Figure 2b. Major urinary protein (MUP, Figures 2c and S13, assigned by intact mass and previously identified by top–down native LESA MS^n^ ^26^) and its proteolysis derivative kidney-fatty acid-binding protein (K-FABP, Figures 2d and S14, assigned by intact mass and previously identified by top–down native LESA MS^n ^^26^) were most abundant within the cortex tissue, with K-FABP being one of the most intense signals overall. The distributions observed for these proteins are consistent with lower resolution native LESA MSI.^26^ The intact homotrimeric complex reactive intermediate deiminase A (RidA, Figures 2e and S15, assigned by intact mass and previously identified by top–down native LESA MS^n^ ^26^) was distributed throughout cortex tissue. In agreement with our observations from native LESA MSI, regucalcin (Figures 2f and S16, assigned by intact mass and previously identified by top–down native LESA MS^n^ ^26^) was found with a more specific distribution than RidA, i.e., to cortex tissue in proximity to the medulla. Both RidA and regucalcin exhibit strong signals within the cortical column compared to MUP, which is indicative of differences in cell specificity. Native nano-DESI provides higher-resolution confirmation that H-FABP (Figures 2g and S17, assigned by intact mass and previously identified by top–down native LESA MS^n^ ^26^) is specific to medullary tissue, as suggested by native LESA MSI.

A nocovalent homodimer previously undetected by native MSI was observed and identified as S100-A6 protein (Figure 2h; see below and Figures S18 and S19 for identification). The S100-A6 homodimer was found within some medullary tissue and an area of the renal pelvis. The monomeric form of this protein is the likely identity of an “unidentified” protein at *m*/*z* 9940 in a previous MALDI MSI study, which shows similar distribution to that observed here.^6^ Another newly identified protein was adipocyte lipid-binding protein (ALBP, Figures 2i and S20; identification in Figure S21). The specificity of the ALBP image is indicative of the adipose tissue surrounding the renal pelvis (renal sinus). Figure 2j shows the distribution of a further newly identified protein by native MSI, phosphatidylethanolamine-binding protein 1 (PEBP1, Figure S22; identification in Figure S23) to be ubiquitous throughout kidney tissues, with absence within blood vessels and the renal pelvis.

Lastly, the distributions of holo-α-globin (α^H^, Figures 2k and S24, assigned by intact mass and previously identified by top–down native LESA MS^n^ ^26^), and the hemoglobin heterodimer (αβ^2H^, Figures 2l and S25) show high specificity to the main blood vessel, and smaller blood vessels within the bulk tissue. The blood vessels are evident as dark regions in images for proteins not found within the blood (Figure S26).

### Spatial Resolution

**Figure 3:**
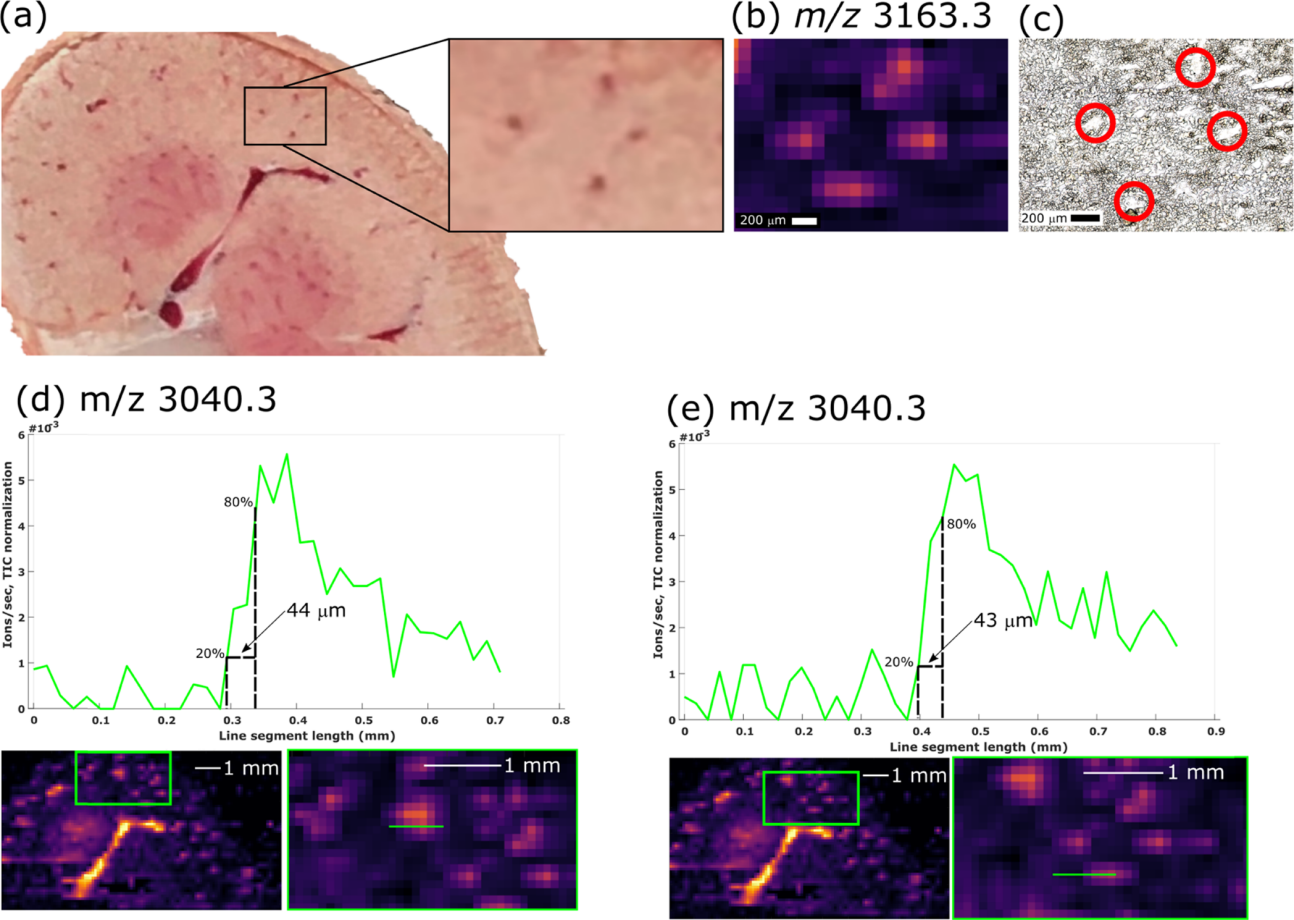
(a) Pattern of four blood vessels was observable on the kidney. The blood vessels (∼200 μm across) are resolved in ion images for *m*/*z* 3163.3 (holo-α globin) with pixel dimensions of (b) 200 μm × 200 μm. (c) Optical image of the tissue section post-analysis; blood vessels are highlighted with the red circles. (d, e) Extracted ion chronograms for *m*/*z* 3040.3 (apo-α-globin) allow approximation of spatial resolution. Tailing of the signal suggests the resolving power is closer to 200 μm.

Prior to this work, the only existing demonstration of native MSI of tissue makes use of native LESA. Previous work with LESA resulted in images featuring pixel size of 1 mm × 1 mm, each pixel representing a discrete sampling point of approximately 0.6 mm diameter. (The discrepancy between sampling area and pixel size is discussed above.) In contrast, nano-DESI is a raster mode technique. The sampling probe used here had a footprint of ∼200 μm in its largest dimension and the native nano-DESI images have a pixel size of 200 μm × 200 μm, accordingly. The Orbitrap Eclipse is a trapping instrument and controls the number of ion charges introduced to either the orbitrap or linear ion trap mass analyzers, by use of automatic gain control (AGC). If the AGC threshold is not reached, injection of ions occurs after a predefined time (“maximum injection time”). For native nano-DESI of kidney, the maximum injection time was set to 500 ms, resulting in a minimum of 2 mass spectra/s. The 200 μm × 200 μm pixels are representative of 10 s of data acquisition since the sample stage was moved at 20 μm/s. Scans were box summed. Signal to noise ratio per pixel is improved because of the summation of signal from multiple scans. The nano-DESI pixels represent a 25× smaller area (0.04 mm^2^) versus published native LESA MSI (1 mm^2^)^16,26^ and vascular features of approximately 200 μm across were resolved (Figure 3a–c). Figure 3a shows blood vessels approximately 200 μm across visible in the photograph of the kidney during sectioning. The ion image of holo-α-globin (*m*/*z* 3163.3, Figure 3b) shows the blood vessels resolved. An optical image of the analyzed tissue section shows the blood vessels highlighted in Figure 3c.

A common approach for estimating nano-DESI resolving power is to determine the distance in which the relative signal intensity changes from 20 to 80%.^21,38^ This estimate requires steep changes in signal on defined features; as such, only vascular features are suitable for this evaluation in the current study because of the intense apo-α-globin signals obtained. Figure 3d,e approximate the resolving power by this method to be ∼44 μm, although this is likely overestimated. Due to the probe footprint, the signal tails and so the resolving power is better approximated to be around 200 μm, which correlates with the size of the resolved features and probe size. Nevertheless, the ability to resolve small vascular features in this first native nano-DESI MSI report shows comparable resolving power to that of raster mode imaging of proteins from tissue, previously only reported under denaturing conditions using DESI,^9^ LMJ-SS,^39,40^ and nano-DESI^11,12^ (typically ≥150 μm laterally).

### Native Nano-DESI MS^n^ Used for In Situ Top–Down Proteomics

**Figure 4:**
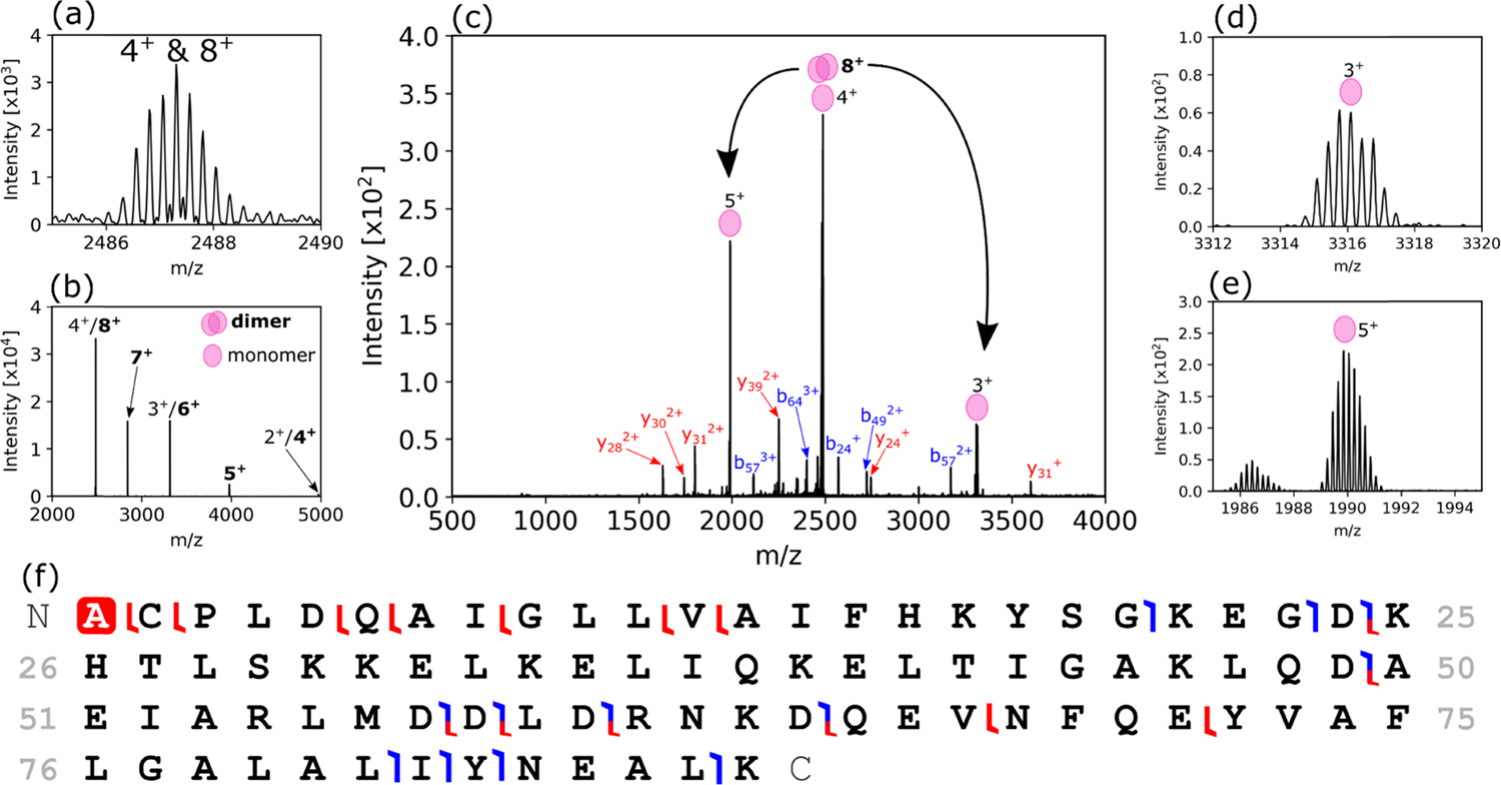
Identification of S100-A6 protein dimer. (a) The precursor ion signal showed overlap of two charge states. (b) PTCR-enabled determination of an 8^+^ charge state obscured by the 4^+^ signal. (c) HCD MS^2^ (NCE = 42%) of *m*/*z* 2487.3 ± 2.5 (average of 148 scans from a raster line scan through the renal pelvis) dissociated the 8^+^ dimer to 5^+^ (d) and 4^+^ and 3^+^ (e) subunits, and produced sequence ions noted in (f).

Native nano-DESI also provides a convenient sampling platform for location-specific tandem mass spectrometry (MS^n^) identification of intact proteins and complexes. MS^n^ spectra can be generated by raster-mode sampling within the region of tissue where the protein signal of interest is most intense, guided by the mass spectrometry images. Raster mode sampling at a slow rate is beneficial to maintaining signal intensity, which otherwise decreases by an order of magnitude after ∼5 min if the probe is set in a static position (see Figure S27). The ability to perform location-targeted MS^n^ is evidence of the reproducibility between serial tissue sections. Figure 4 shows the identification of an intact homodimer, protein S100-A6 (∼18.88 kDa), which was new to our in situ analyses of proteins in the rat kidney (see also above). The most intense dimer signal (8^+^) overlapped with the 4^+^ charge state of the monomer (Figure 4a), but this was difficult to ascertain with high-resolution MS alone. PTCR was used to confirm the presence of the 8^+^ dimer (Figure 4b), with a series of charge-reduced ions being produced. The intensity distribution, which typically exhibits lower intensity for successively lower charge states, was indicative of overlap between monomer and dimer signals. HCD MS^2^ of ions with *m*/*z* 2487.3 ± 2.5 (Figure 4c) confirmed the presence of the dimer; monomer subunits were detected as dissociation products in 5^+^ (Figure 4d), 4^+^, and 3^+^ (Figure 4e) charge states along with sequence fragments enabling identification (Figure 4f).

Small molecules bound to proteins can be investigated by a similar approach. Figure S28 shows HCD MS^2^ of the 9^+^ charge state of hemoglobin dimer αβ^2H^, resulting in monomer, polypeptide sequence fragments, and heme ions. Subsequent HCD MS^3^ of the heme ions provided a characteristic product ion. The same strategy was used to identify somatic cytochrome C, a protein in which the heme group is covalently bound to the protein (Figure S29). HCD MS^2^ of the precursor ion at approximately *m*/*z* 2022.8^6+^ provided two major product ions indicating the dissociation of both covalent bonds between heme and the cysteine residues. HCD MS^3^ of the singly charged product (*m*/*z* 617.2) confirmed its identity as heme. Notably, it featured an additional proton compared to the noncovalently bound heme from hemoglobin complexes, as a result of the covalent bond cleavage.^41^

**Figure 5:**
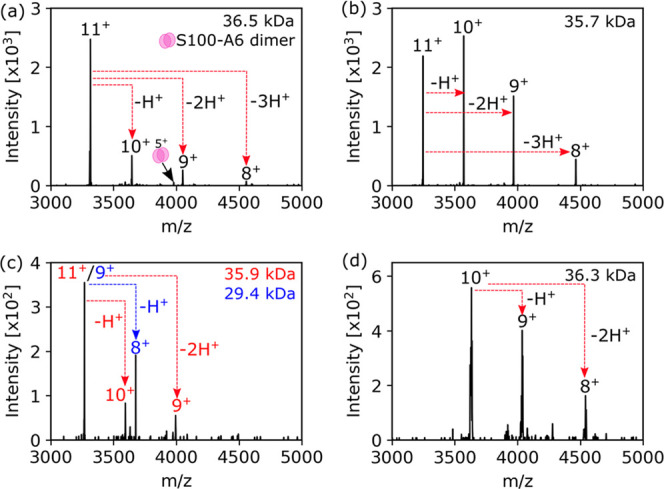
PTCR-enabled charge state determination for low-abundance proteins, which cannot be isotopically resolved by high-resolution MS. (a) *m*/*z* 3315.94^11+^ (36.5 kDa), the 5^+^ charge state of S100-A6 is also detected due to overlap of the (unknown) 11^+^ precursor with the (S100-A6 dimer) 6^+^ precursor. (b) *m*/*z* 3247.07^11+^ (35.7 kDa), (c) *m*/*z* 3268.25^9+/11+^ (29.4/35.9 kDa) indicated the presence of two overlapping signals within the isolation window. (d) *m*/*z* 3632.97^10+^ (36.3 kDa).

The combination of low signal intensity, spectral overlap, and high molecular weight (MW) (>30 kDa) still poses a significant challenge for in situ top–down identifications. Here, we have made use of PTCR to provide molecular weights for a series of low-abundance proteins with MW approximately 36 kDa (Figure 5) that were not determinable with high-resolution MS. Figure 5c shows an example of two overlapping proteins at *m*/*z* 3268 that would produce convoluted MS^2^ spectra but are differentiated by PTCR.

Adipocyte lipid-binding protein (ALBP, 14.7 kDa) was identified by native nano-DESI HCD MS^2^. Inspection of the ion image for *m*/*z* 2093^7+^ showed the most intense signal in the renal pelvis; therefore, nano-DESI MS^2^ was performed in this region. Figure S21a shows the HCD MS^2^ spectrum, with Figure S21b,c showing spectral details. The intact mass and sequence fragments were consistent with an isoform of ALBP (Figure S21d,e, Uniprot Q5XFV4).

The identity of signals at *m*/*z* 2589.0^8+^ was tentatively assigned by intact mass alone (PEBP1, ∼20.7 kDa) using PTCR but was challenging to confirm by fragmentation (Figure S23). As the protein was homogeneously distributed and of low relative signal intensity, the strategy was to run line scans across the kidney cortex tissue. Optimization remains difficult in this situation due to low signal intensity per scan and represents the practical limit for top–down experiments of this manner. Still, two isotopically resolved main sequence product ions were detected by CID MS^2^ after two line scans through cortex tissue. Both fragments were the result of cleavage at Asp-Pro in the sequence of PEBP1, which have the highest propensity for cleavage for native proteins under collisional activation.^42^

## Conclusions

Adaptation of nano-DESI for native MSI resulted in considerable improvements in the ability to resolve small tissue features and tissue morphology compared with previous reports of native MSI using LESA. For proteins detected in rat kidney by native nano-DESI MSI that have previously been reported with native LESA MSI, there is strong agreement between the low-resolution LESA images reported and these new higher-resolution nano-DESI images, but now with the ability to resolve finer details of kidney anatomy. Location-targeted native nano-DESI MS^n^ enabled novel proteins to be identified in situ, including the noncovalent homodimer S100-A6. The largest protein detected was the RidA trimer (42.6 kDa). PTCR shows promise as a technique for deconvoluting overlapping signals in low-resolution mass spectra and determining the MW of low-intensity proteins in in situ top–down experiments.

Native MSI can now be applied to studies requiring finer features of tissue sections to be resolved, potentially allowing protein–ligand complexes to be studied with greater tissue-type specificity, e.g., in pharmaceutical development. A route to even higher spatial resolution includes more finely produced solvent capillaries, but would likely correspond with weaker signal intensity. An oversampling approach could be used, but this would need to be validated as suitable for native protein analysis and may further reduce already weak ion signals. Alternatively, microscopy-assisted resolution improvements demonstrated by Lin et al. offer the possibility of improvement through software approaches.^12^

## Supporting Information Available

Photographs of nano-DESI source and control electronics (Figures S1 and S2); nano-DESI line scan mass spectra from rat kidney (Figure S3); native nano-DESI of protein standards and estimates of LOD (Figures S4–S6); single-scan native nano-DESI mass spectra (Figure S7); comparison of native nano-DESI and native LESA mass spectra (Figure S8); nano-DESI MS with organic solvent systems (Figures S9–S11); ion images from kidney section replicates (Figure S12); ion images for multiple charge states of MUP, K-FABP, RidA, regucalcin, H-FABP, S100-A6, ALBP, PEBP1, α^H^, and αβ^2H^ (Figures S13–S18, S20, S22, S24, and S25); composite ion image (Figure S26); tandem MS of S100-A6 dimer, ALBP, PEBP1, αβ^2H^, and cytochrome C (Figures S19, S21, S23, S28, and S29); and assessment of signal duration for static probe (Figure S27) (PDF)
