# Supplementary material for: Native Mass Spectrometry Imaging of Proteins and Protein Complexes by Nano-DESI
Source: Anal Chem. 2021 Mar 4;93(10):4619–27. doi: 10.1021/acs.analchem.0c05277 (PMC8034770; doi:10.1021/acs.analchem.0c05277)
Supplement: Supplementary file 1 — ac0c05277_si_001.pdf [file ac0c05277_si_001.pdf]

Supporting information for "Native mass spectrometry imaging of proteins and protein complexes by nano-DESI."

Oliver J. Hale and Helen J. Cooper*

School of Biosciences, University of Birmingham, Edgbaston B15 2TT, U.K.

*To whom correspondence should be addressed: h.j.cooper@bham.ac.uk

Contents	Page
Figure S1: Photographs of the ion source setup.	S3
Figure S2: Photograph of control electronics for nano-DESI 2D stage.	S4
Figure S3: Example nano-DESI line scans from rat kidney.	S4
Figure S4: Protein standards analyzed by native nano-DESI	S5
Figure S5: Estimate of the limit of detection (LOD)	S6
Figure S6: Method for LOD approximation	S7
Figure S7: Native nano-DESI mass spectra, single-scans	S8
Figure S8: Mass spectra from native LESA and native nano-DESI	S9
Observations from nano-DESI with organic solvent-based systems	S10
Figure S9: Nano-DESI MS with organic solvent systems (High energy desolvation)	S11
Figure S10: Nano-DESI MS with organic solvent systems (Low energy desolvation)	S12
Figure S11: Nano-DESI MS with organic solvent system. (Renal pelvis)	S13
Figure S12: Ion images for three kidney replicates	S14
Figure S13: Ion images for multiple charge states of MUP	S15
Figure S14: Ion images for multiple charge states of K-FABP	S15
Figure S15: Ion images for multiple charge states of RidA	S15
Figure S16: Ion images for multiple charge states of regucalcin	S15
Figure S17: Ion images for multiple charge states of H-FABP	S15
Figure S18: Ion images for multiple charge states of S100-A6	S16
Figure S19: HCD MS^2 of S100-A6 dimer 7^+	S16
Figure S20: Ion images for multiple charge states of ALBP	S16
Figure S21: Identification of ALBP by HCD MS^2.	S17
Figure S22: Ion images for multiple charge states of PEBP1	S17
Figure S23: Identification of PEBP1 by CID MS^2.	S18
Figure S24: Ion images for multiple charge states of α^{H}	S18
Figure S25: Ion images for multiple charge states of $\alpha\beta^{2H}$	S18
Figure S26: Composite ion image showing blood vessels amongst bulk tissue	S19
Figure S27: Assessment of signal duration for static probe	S19
Figure S28: HCD MS^n of $\alpha\beta^{2H\ 9+}$	S20
Figure S29: HCD MS^n of cytochrome C	S20
References	S21

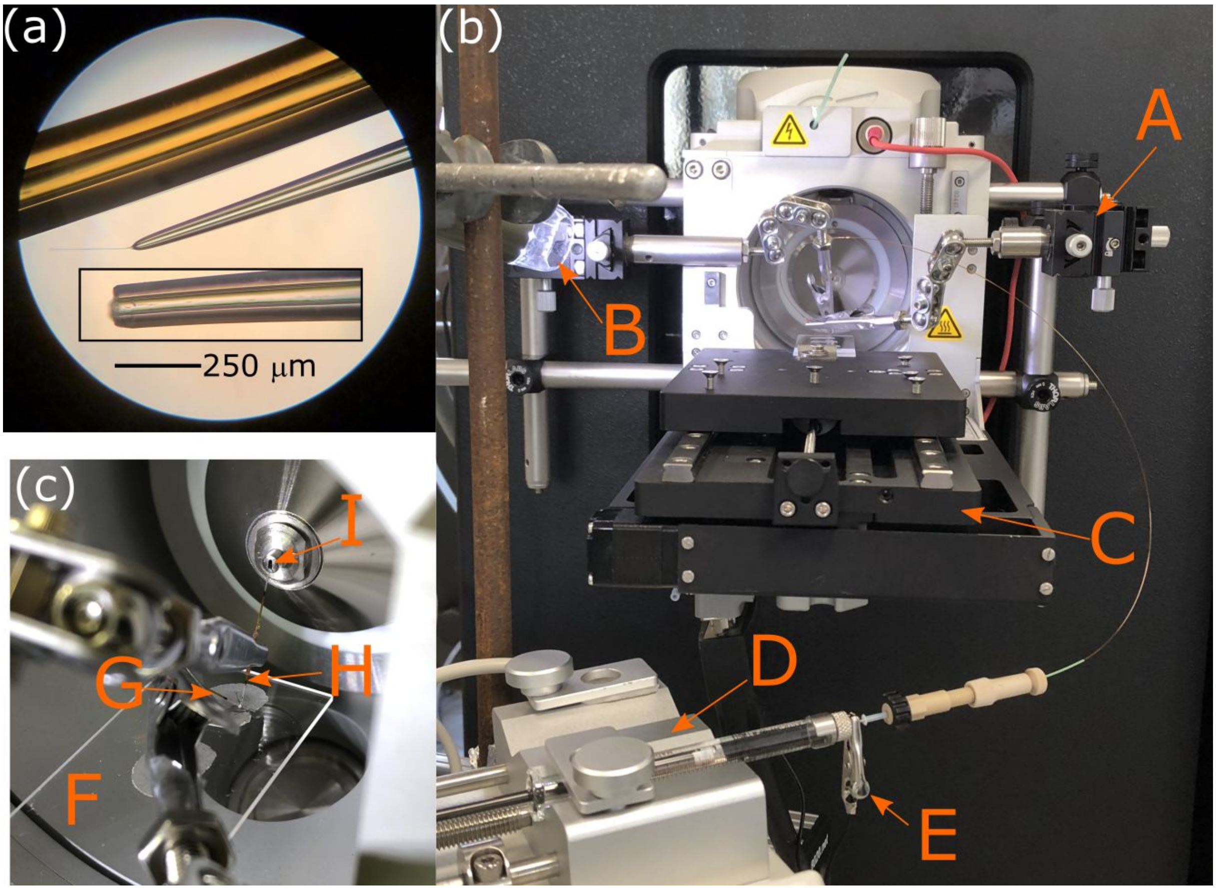

Figure S1: (a) tapering of the flame-pulled (colorless) fused silica capillary viewed under a 10x microscope lens. The capillary was then cut approx. 2 mm from the point to produce a tip ~50 μm I.D. and 100 μm O.D (see inset). The amber capillary is as bought from the supplier prior to flame-pulling, for comparison (275 μm O.D. 75 μm I.D.) (b) photograph of the ion source. Labels for components: **A;** xyz-manipulator, **B;** camera, **C;** programmable xy-stage, **D;** syringe pump, **E;** high voltage connection. (c) positioning of the fused silica capillaries on a sample: **F;** slide surface, **G;** primary capillary, **H;** sampling capillary, **I;** mass spectrometer inlet.

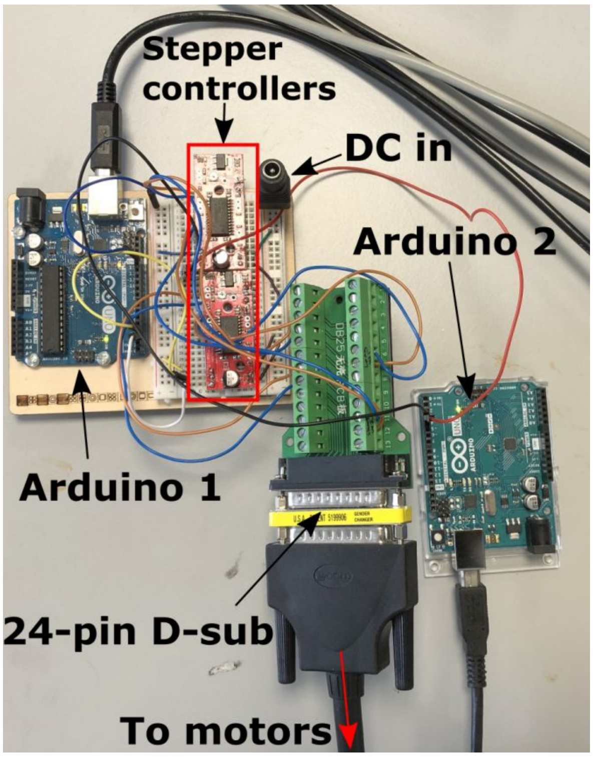

Figure S2: Custom control electronics for the XY-stage motors. One Arduino Uno coupled to one stepper controller was used for each axis. The stepper controllers were set to 1/8 microstepping. A 5 V/2 A DC power supply was used to power the motors (5 V, 0.49 A per winding, 2 windings per motor). The Arduinos were sent commands from the Arduino software serial monitor.

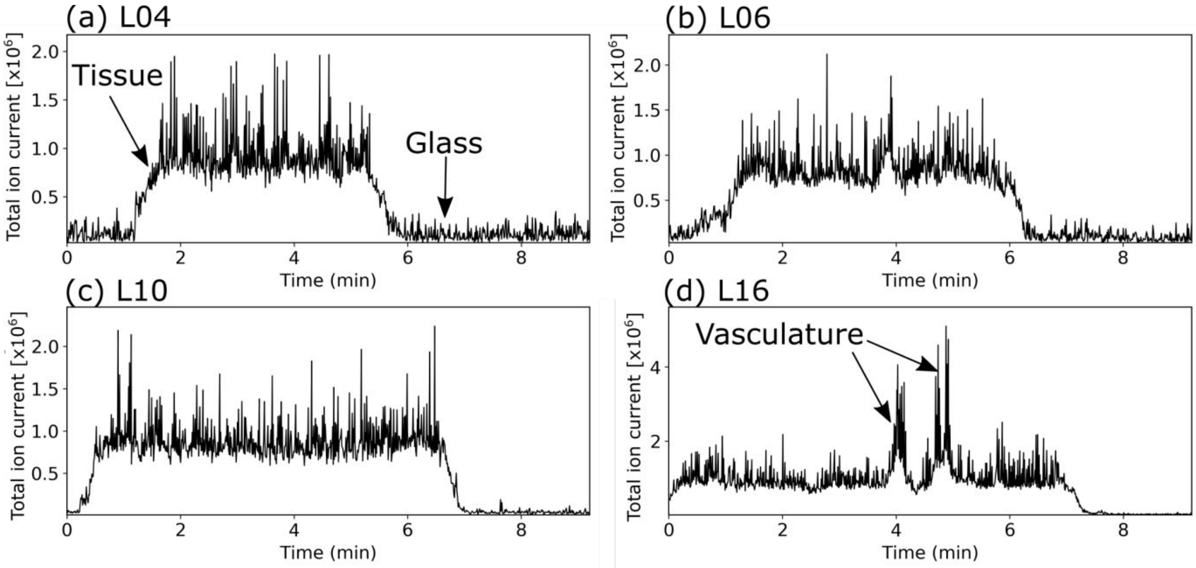

Figure S3: Example line scans showing total ion current for line 4, 6, 10 and 16 (a – d respectively) for the rat kidney. Peaks in intensity are often due to vasculature, especially in (d) where a large blood vessel was crossed.

Native nano-DESI of protein standards.

Initial investigations focused on whether native-like mass spectra could be produced with the nano-DESI source. Using LESA, we have shown that proteins are not refolded during extraction from a surface, rather native mass spectra are representative of the protein conformation on the surface.[1] Native nano-DESI analysis of dried monomeric protein droplets produced protein ion signals distributed over a narrow charge state range, indicative of folded proteins with limited solvent-accessible surface area. **Figure S4a** shows the native nano-DESI mass spectrum of HEWL revealing charge state 7^+ and 8^+, typical of its analysis by ESI-based native MS. **Figure S4b** shows the native nano-DESI mass spectrum of myoglobin dominated by three charge states of the holo-protein where heme remains non-covalently bound. Minor peaks of the apo-protein were also detected. Native nano-DESI of ovalbumin produced two intense charge states (12^+ and 13^+) relating to the monomeric conformation (**Figure S4c**).

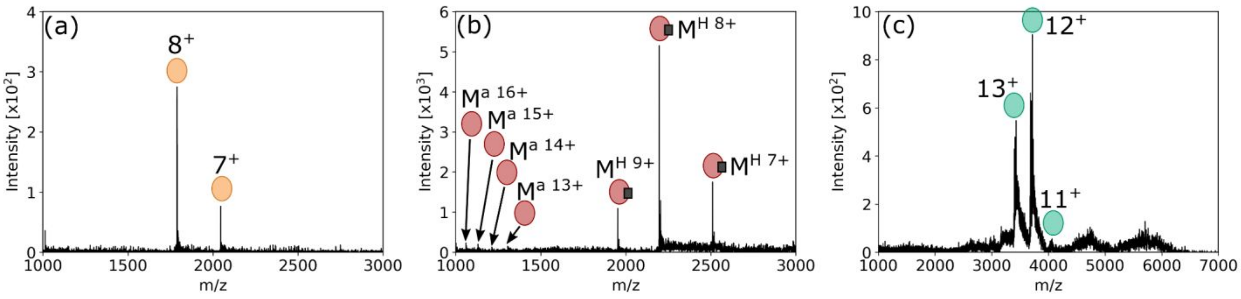

Figure S4: Monomeric proteins analyzed by static nano-DESI using 200 mM ammonium acetate from a dried droplet on a glass slide: (a) HEWL (160 pmol on the slide, average of 100 scans); (b) myoglobin (670 pmol on the slide, average of 140 scans) showing predominantly heme-bound (holo = M^H) ions and weak signals for the denatured protein (apo = M^a); and (c) ovalbumin (420 pmol on the slide, average of 100 scans).

Limit of detection

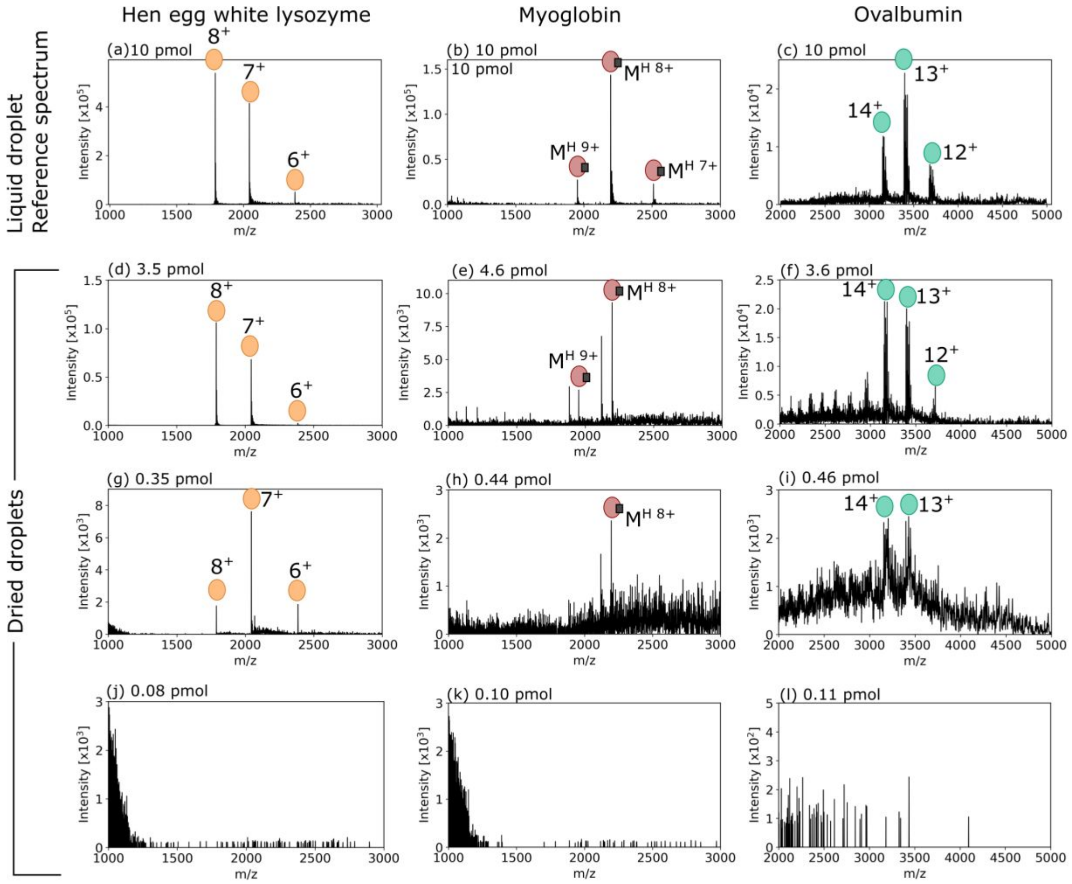

Figure S5: Estimating the limit of detection using protein standards.

1 µL droplets of 10 µM (a) HEWL, (b) myoglobin and (c) ovalbumin in 200 mM ammonium acetate were deposited directly onto the nano-DESI probe to produce reference native mass spectra.

For each protein standard, 0.2 µL of solutions with protein concentrations 100 pmol/µL, 10 pmol/µL, and 1pmol/µL in 200 mM ammonium acetate were dried onto glass substrates (n=5 for each concentration). The area sampled, and therefore amount consumed, during a nano-DESI line scan analysis across the dried protein spots were calculated as described in Figure S6.

The native nano-DESI mass spectra obtained from dried 100 pmol/µL protein spots of (d) HEWL (total amount ~3.5 pmol), (e) myoglobin (total amount ~4.6 pmol) and (f) ovalbumin (total amount ~3.6 pmol). The native nano-DESI mass spectra obtained from dried 10 pmol/µL protein spots of (g) HEWL (total amount ~0.35 pmol), (h) myoglobin (total amount ~0.44 pmol) and (i) ovalbumin (total amount ~0.46 pmol). The native nano-DESI mass spectra obtained from dried 1 pmol/µL protein spots of (j) HEWL (total amount ~0.08 pmol), (k) myoglobin (total amount ~0.10 pmol) and (l) ovalbumin (total amount ~0.11 pmol).

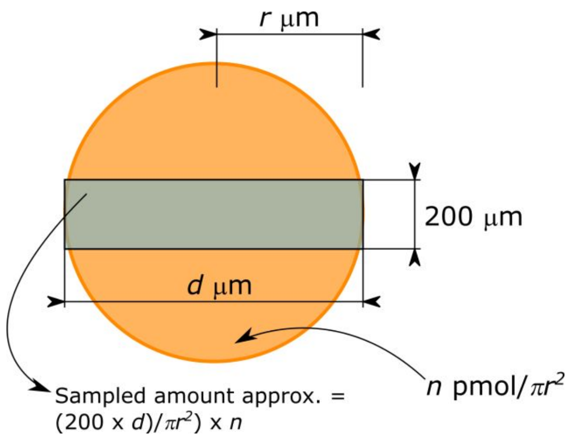

Figure S6: Method for approximation of the LOD from protein standards deposited as dried droplets on a glass slide. The average LOD for HEWL, myoglobin and ovalbumin were estimated to be 0.36, 0.45 and 0.42 pmol respectively from 5 replicate dried droplets of 2 pmol total deposit. The estimate is based on the area sampled by the nano-DESI probe. Several assumptions were made: the dried droplet being circular, homogenous distribution of the analyte, sampling through the center point of the circle and negligible difference due to the rectangular area.

E.g. For **Figure S5(g)**:

d = 1.47 mm (determined by the width of the signal in the TIC chronogram x probe movement speed)

2 pmol HEWL evenly distributed over a circular area = 1.70 mm^2.

Sampled area = 1.47 x 0.2 = 0.294 mm^2

Approx. 17.3% area sampled = 2 pmol x 0.173 = 0.35 pmol consumed. Signals were detected in the mass spectrum for this amount.

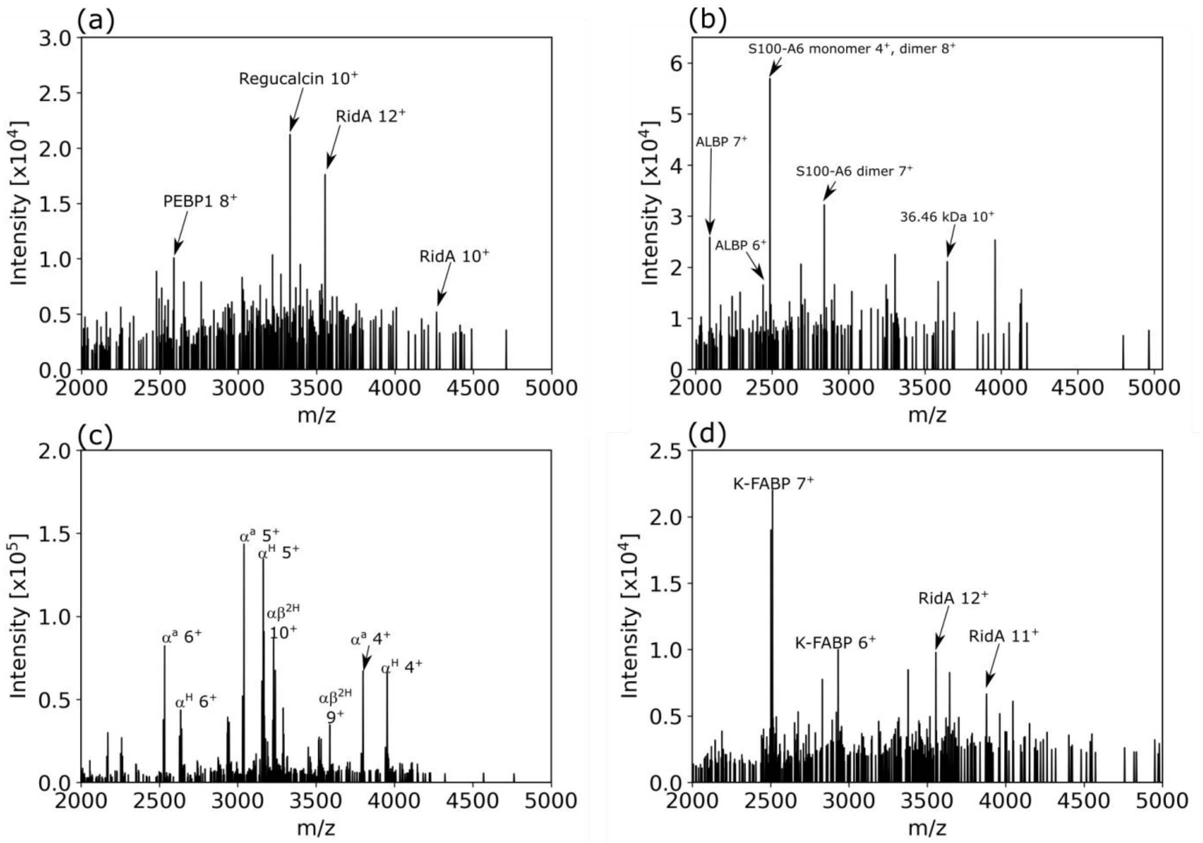

Figure S7: Native nano-DESI mass spectra for single scans from different regions of the kidney. (a) inner cortex, (b) renal pelvis, (c) blood vessel, (d) outer cortex. Pixels in ion images are the average of between 10 and 20 single scans to the benefit of signal-to-noise ratio.

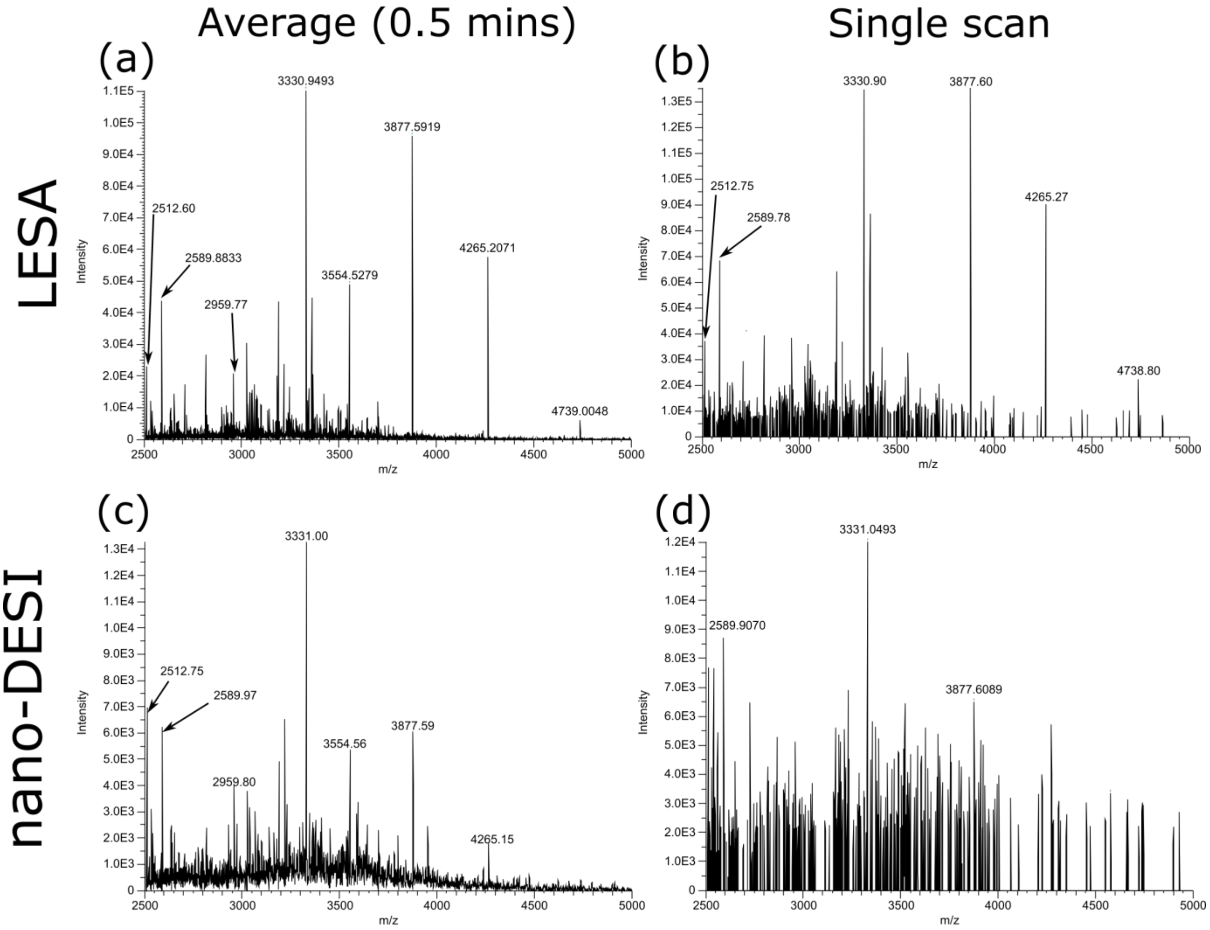

Figure S8: representative mass spectra from the renal cortex for LESA (a, b) and nano-DESI (c, d). Average (a, c; all scans within a 0.5 min window) and single scan (b, d) show that native LESA generally results in between 1-2 orders of magnitude more intense signal than nano-DESI.

The comparison of mass spectra obtained by LESA and nano-DESI is not straightforward. Each LESA pixel was sampled with 5 μL solvent, almost 17x greater volume than each pixel for nano-DESI (~300 nL, 1.9 μL/min).

LESA pixels have an area of 1 mm^2. The actual sampled area for LESA is equivalent to a circular area under the pipette tip, approx. 0.6 mm diameter, which leads to approximately 7x greater than nano-DESI per pixel (~0.28 mm^2 versus 0.04 mm^2). This suggests sample concentration could be lower for LESA. LESA sample consumption during ionization is <200 nL/min, determined from sprays lasting 30 mins or longer for 5 μL samples. Yet LESA signal intensity is approx. 10x higher per scan. We suggest that the much narrower diameter ESI emitter used for LESA (2.5 μm) than nano-DESI (~75 μm) results in more efficient protein desolvation and contributes to the improved signal intensity for LESA.[2]

Observations from nano-DESI with organic solvent-based systems.

Acetonitrile (ACN)-based solvent systems containing formic acid (FA) have been used for protein analysis by nano-DESI previously[4-6]. To demonstrate the effect of denaturants like organic solvents and acids, nano-DESI linescans were performed with ACN and methanol-based solvent systems on cortex rat kidney tissue. High energy desolvation conditions, the same as used for ammonium acetate-based nano-DESI imaging reported in this article, and low energy desolvation conditions were investigated (Figures S9 and S10 respectively).

The highest ACN content (i.e. 80%) favored lipid extraction and ionization. Highly charged protein signals in the low mass range were attributable to small proteins like cytochrome C and apo-α-globin (S9a, S10a) and there were no discernible protein ions signals in the higher mass range (S9b, S10b).

65% ACN/0.1% FA provided a relative increase in apo-α/β-globin signals (S9c, S10c), especially in the high mass range (S9d) under high energy conditions, but not under low energy conditions (S10d).

50% ACN/0.1% FA further increased apo-α/β-globin signals (S9e, S10e). 50% ACN/0.1% FA under high energy desolvation conditions showed signals for monomers and dimers of RidA indicative of dissociation. Under low energy conditions (S10f) the intact trimer was detected but with a broader charge state envelope and higher charge states (13^+-18^+) than under native conditions (9^+-12^+). Under native conditions, 85 V source CE does not dissociate the RidA complex (see Figure 1d). $\alpha\beta^{2H}$-globin dimers were not detected in 50% ACN/0.1% FA.

Where the organic solvent was methanol, lipids and apo-α/β-globin were readily detectable under both desolvation conditions in the low mass range (S9g, S10g)

Protein ion signals that were abundant in the native mass spectra e.g. K-FABP were not detected, even considering higher charge states of those proteins.

Figure S11 shows mass spectra from nano-DESI linescans of renal pelvis kidney tissue. The spectra reveal that the S100-A6 dimer did not remain intact in 50% ACN conditions even under low energy desolvation conditions.

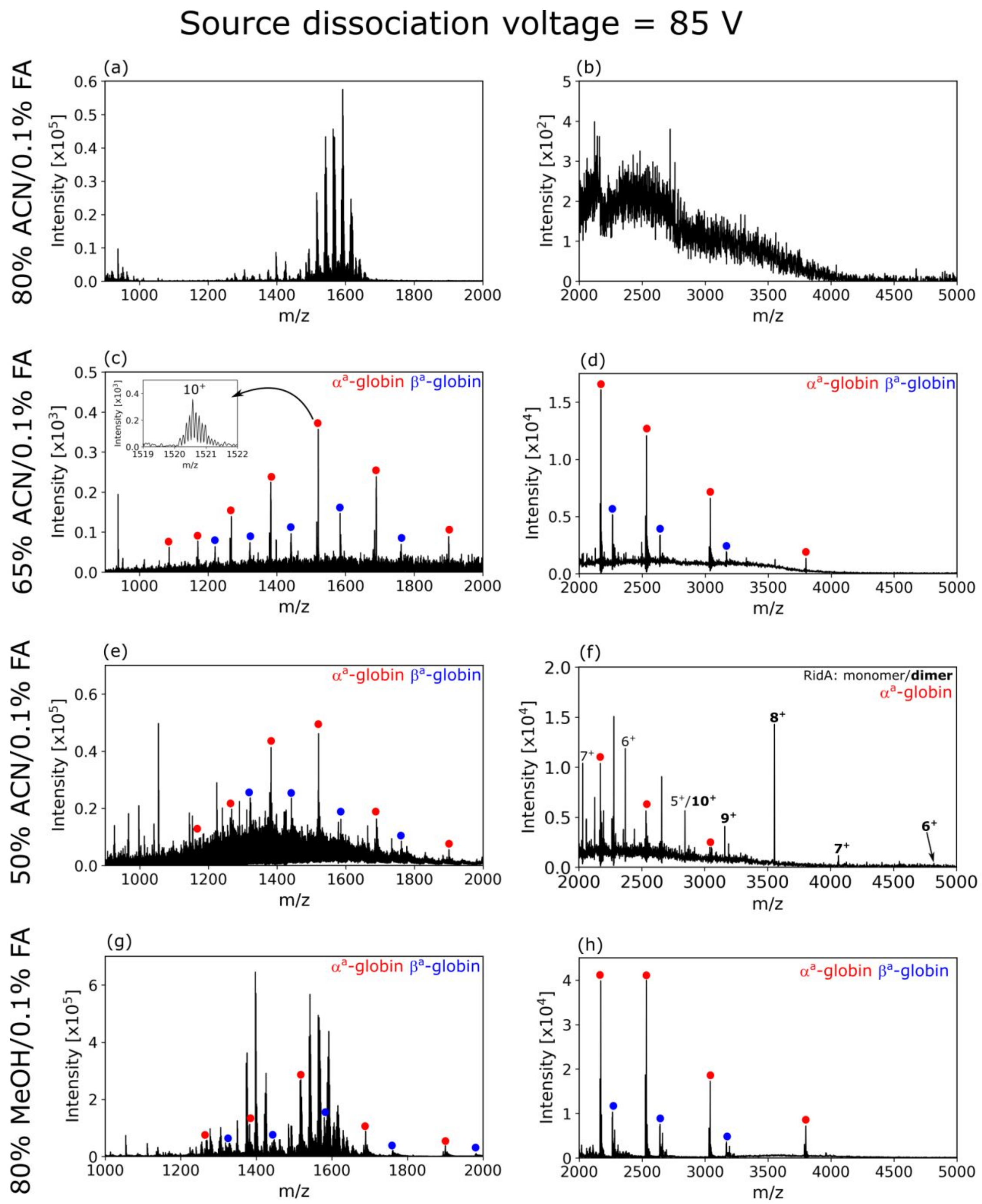

Figure S9: Mass spectra for nano-DESI of proteins from rat kidney cortex with organic solvents under high energy desolvation conditions (i.e. source dissociation voltage = 85 V). (a) 80% acetonitrile favored lipid extraction with the low mass range featuring weak apo-α-globin signals and (b) no protein ion signals in the high mass range. (c) 65% ACN provided a relative increase in apo-α/β-globin signal intensity in the low mass range and revealed low charge state signals in the high mass range (d). 50% ACN further increased apo-α/β-globin signals. (f) RidA monomers and dimers indicative of trimer dissociation were detected in the high mass range. 80% MeOH/0.1% FA spectra were dominated by singly charged and apo-α/β-globin signals. Each spectrum represents approx. 0.24 mm^2 of tissue.

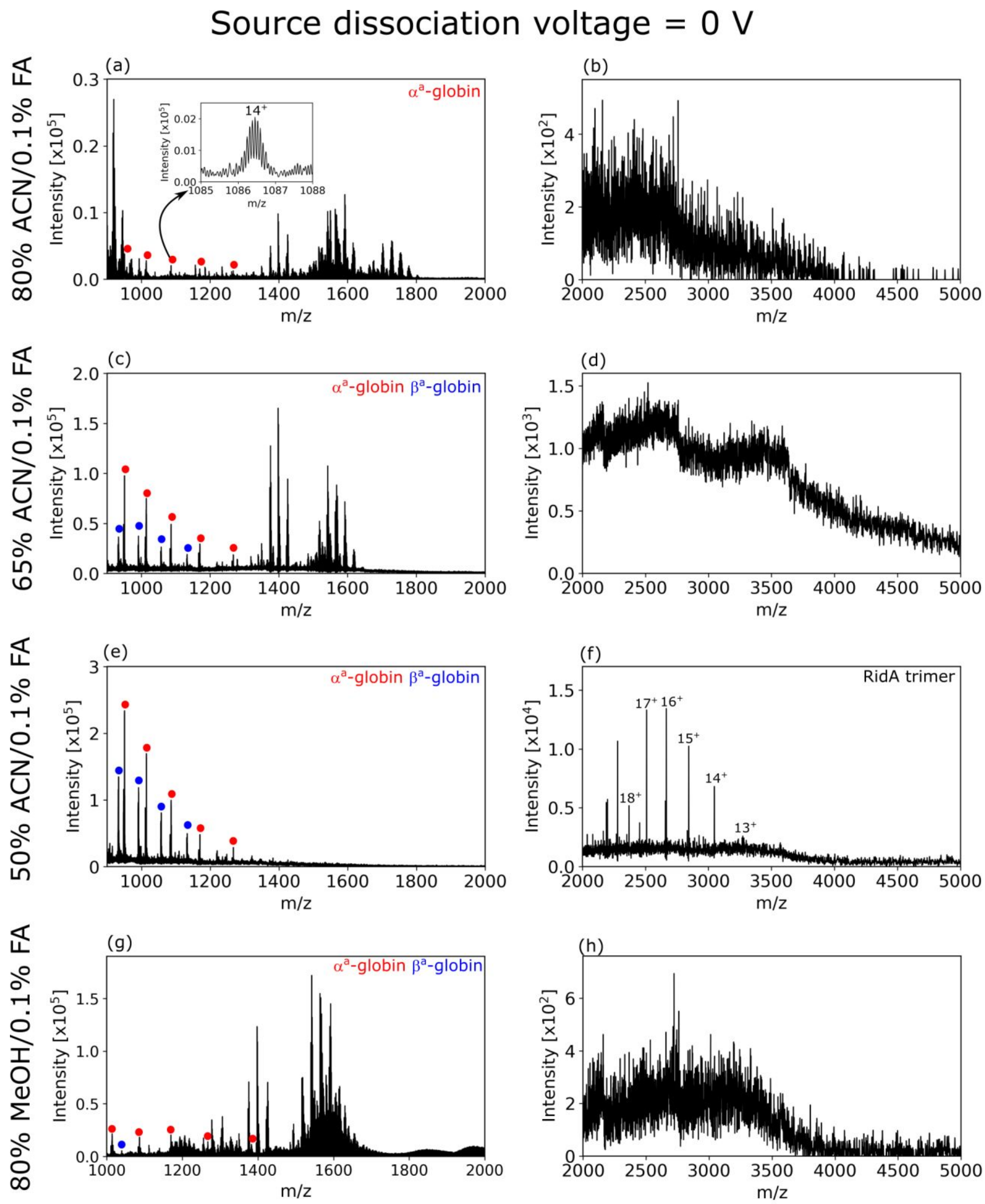

Figure S10: mass spectra from nano-DESI analysis of rat kidney cortex with organic solvents under low energy desolvation conditions (i.e. source dissociation voltage = 0 V). Lipid-related signals were abundant in the low mass range (a) for 80% ACN/0.1% FA. Only apo-α-globin signals were recognizable for proteins, and protein ion signals were absent from the high mass range (b). Highly charged protein ions, especially apo-α/β-globin subunits were abundant under more aqueous conditions; 65% ACN/0.1% FA (low mass, c; high mass, d) and 50% ACN/0.1% FA (low mass, e). Interestingly, the RidA trimer was detected intact in 50% ACN/0.1% FA (f) but with a broader charge state envelope and higher charge states than under native conditions. $\alpha\beta^{2H}$-globin dimers were not detected. 80% MeOH/0.1% FA featured abundant lipid signals and lower intensity signals for apo-α/β-globin Each spectrum represents approx. 0.24 mm^2 of tissue.

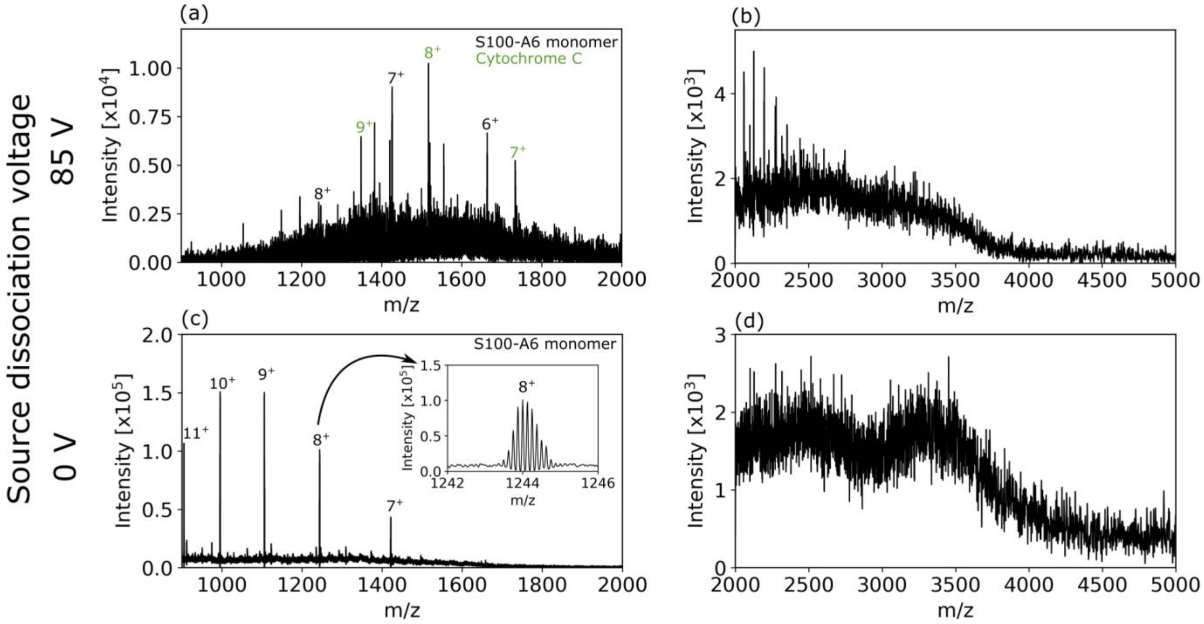

Figure S11: mass spectra from nano-DESI analysis of the renal pelvis with 50% ACN/0.1% FA under high energy (a, b) and low energy (c, d) desolvation conditions. Dimers of S100-A6 protein were not detected under either condition despite high abundance of monomers.

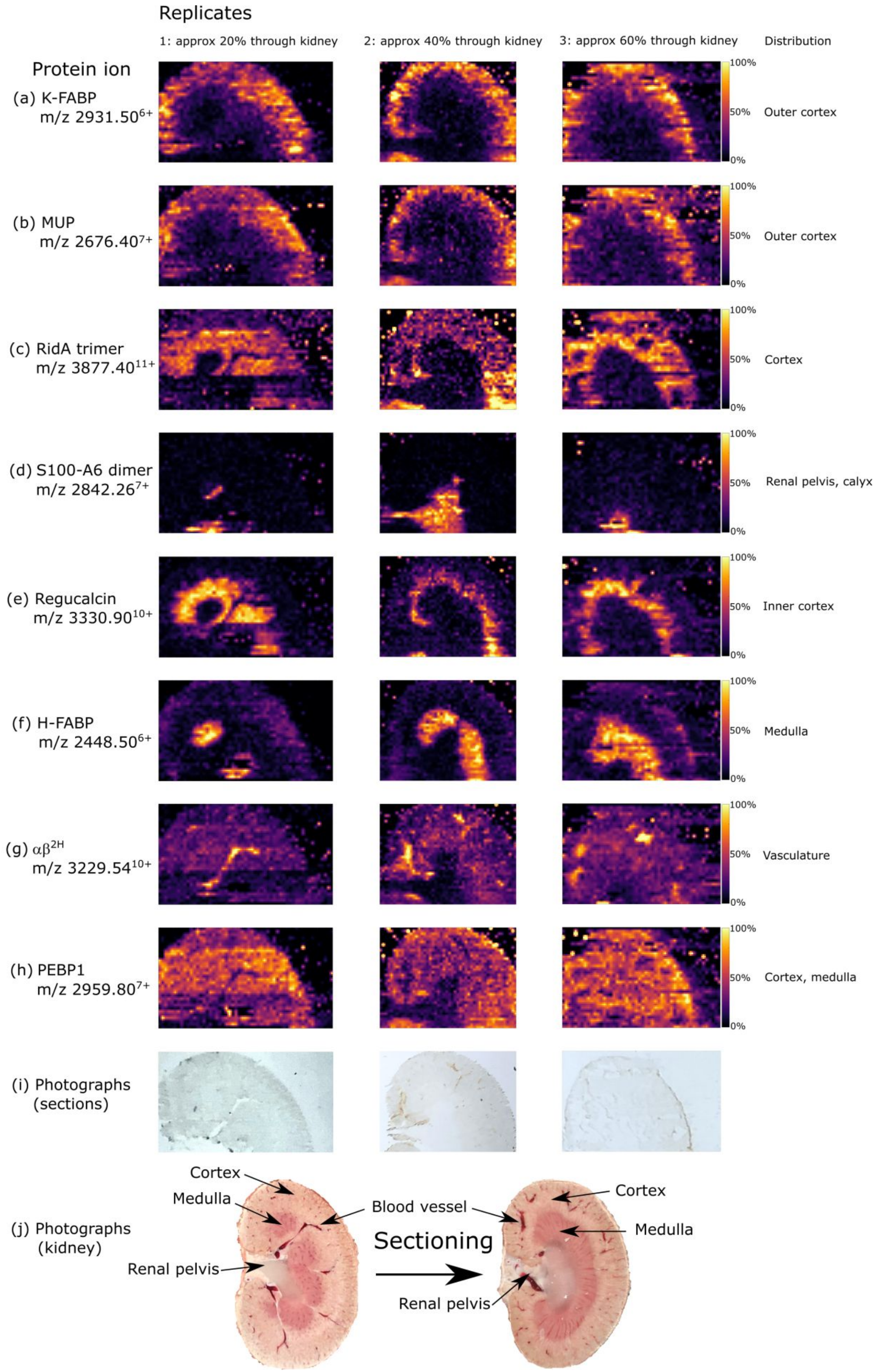

Figure S12: Ion images for three replicate kidney sections showing reproducible detection of folded proteins and intact protein assemblies specific to regions of kidney tissue. Note that replicates 2 and 3 were obtained from sections deeper into the kidney than for replicate 1 thus there was greater abundance of medulla tissue. The evolution of the tissue during sectioning is shown in (j).

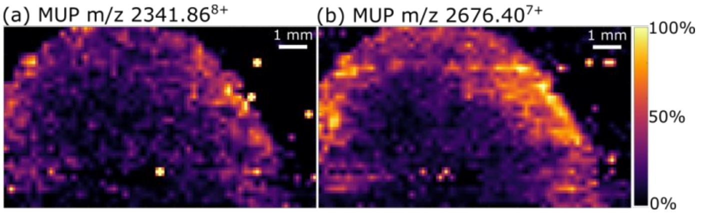

Figure S13: Ion images for two charge states of MUP display similar distributions, with highest abundance in the renal cortex.

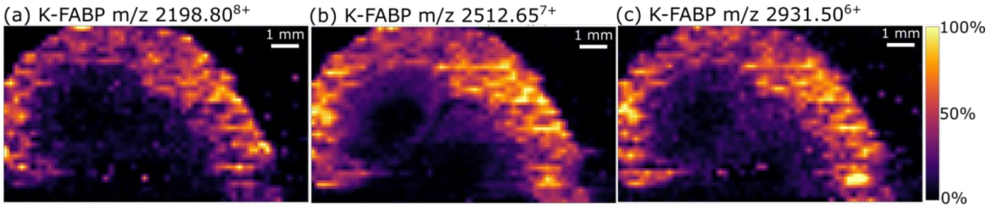

Figure S14: Ion images for three charge states of K-FABP allow confidence in its distribution in the renal cortex.

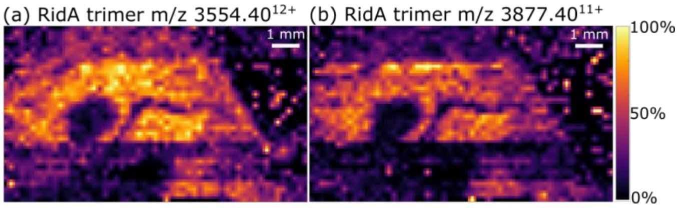

Figure S15: Ion images for two charge states of RidA show its broad distribution in cortex tissues, and absence in the medulla and blood vessels.

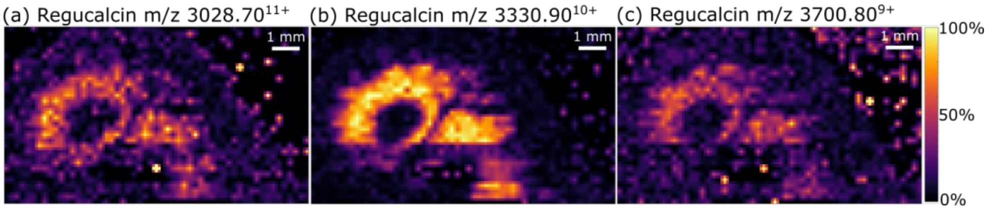

Figure 16: Ion images for three charge states of regucalcin show that it is located within inner cortex tissue and absent from the medulla and blood vessels.

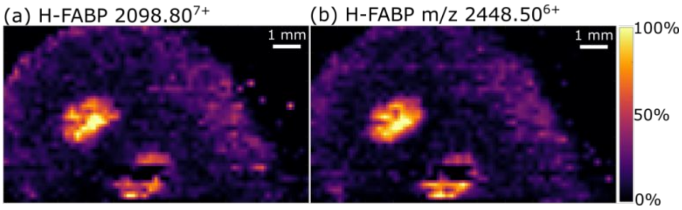

Figure S17: Ion images for two charge states of H-FABP show that it is most abundant in the medulla tissue.

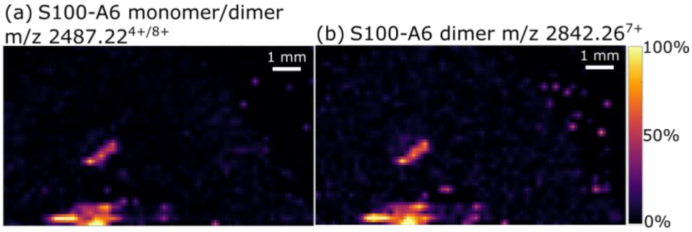

Figure S18: ion images for S100-A6 (a) monomer/dimer overlapping $4^+/8^+$ charge states and (b) dimer 7^+ show specific distribution adjacent to medullary tissue and within the renal pelvis.

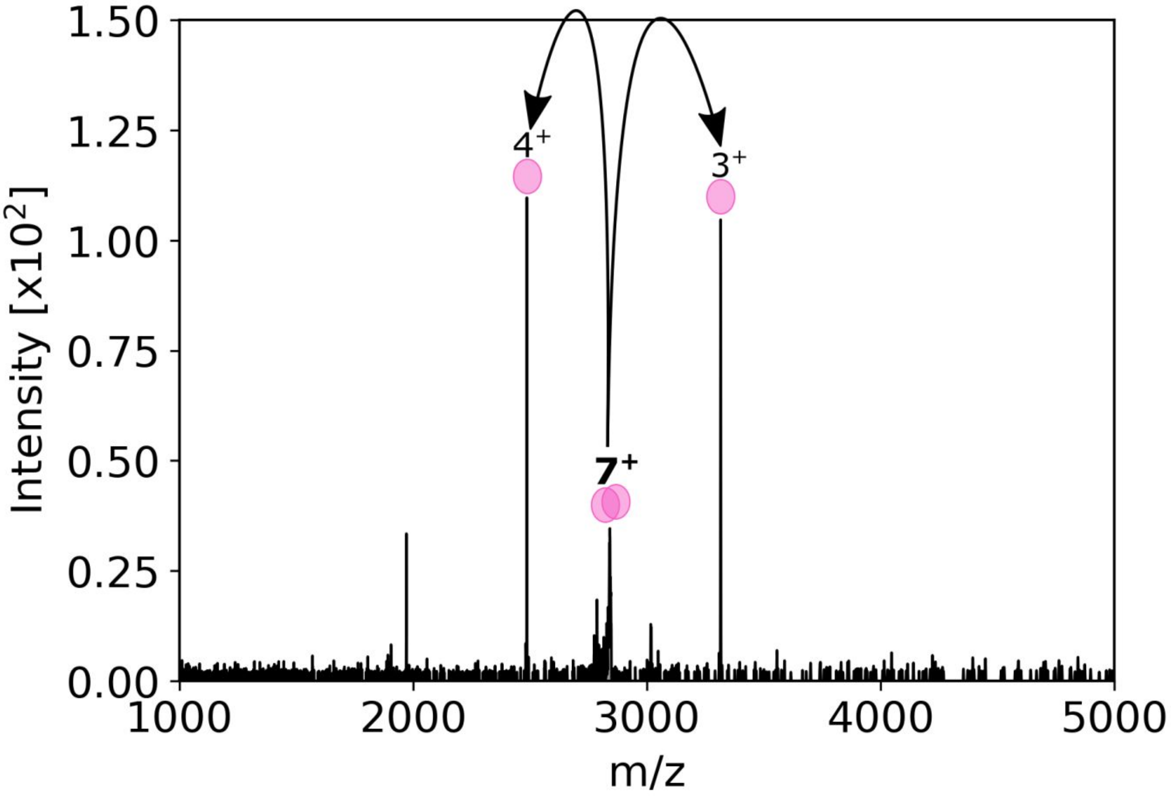

Figure S19: HCD MS^2 (NCE = 30%) of the 7^+ dimer of S100-A6 protein to monomer subunits.

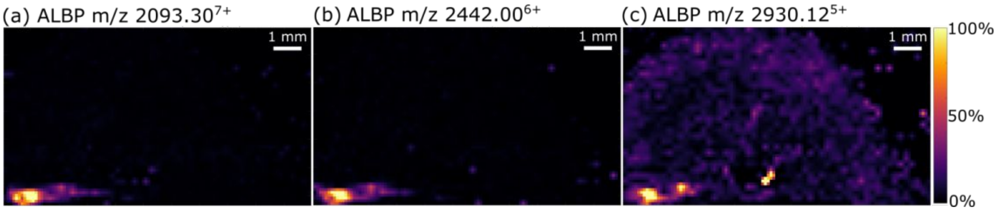

Figure S20: Ion images for three charge states of ALBP show its very specific distribution within the renal pelvis.

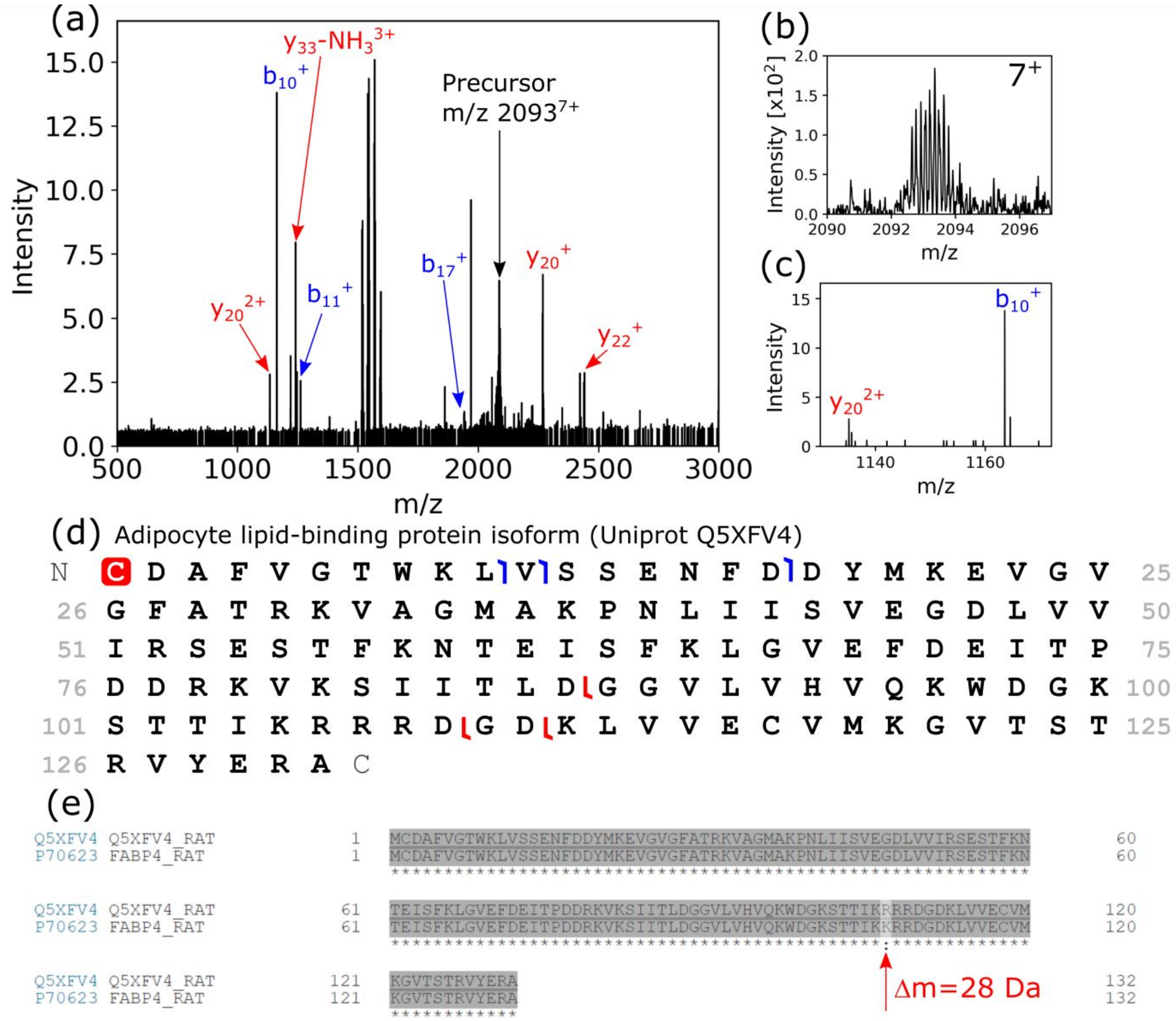

Figure S21: Identification of ALBP; (a) HCD MS^2 (NCE = 44%) spectrum of the precursor signal at m/z 2093^{7+} (14637.83 Da). (b) and (c) show details of precursor and select product ions respectively. (d) The matched amino acid sequence for the ALBP isoform (Uniprot Q5XFV4) with a mass difference of 15 ppm. (e) Sequence alignment of two ALBP isoforms showed substitution of K for R resulting in a Δm = 28 Da to the isoform P70623.

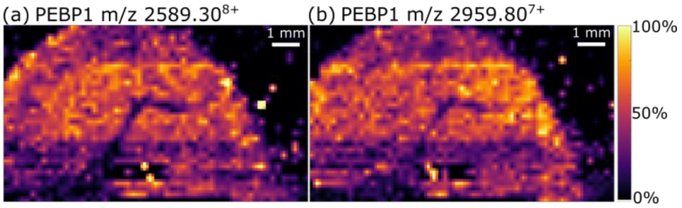

Figure S22: Ion images for two charge states of PEBP1 show its largely homogenous distribution throughout kidney tissues, and absence in blood vessels.

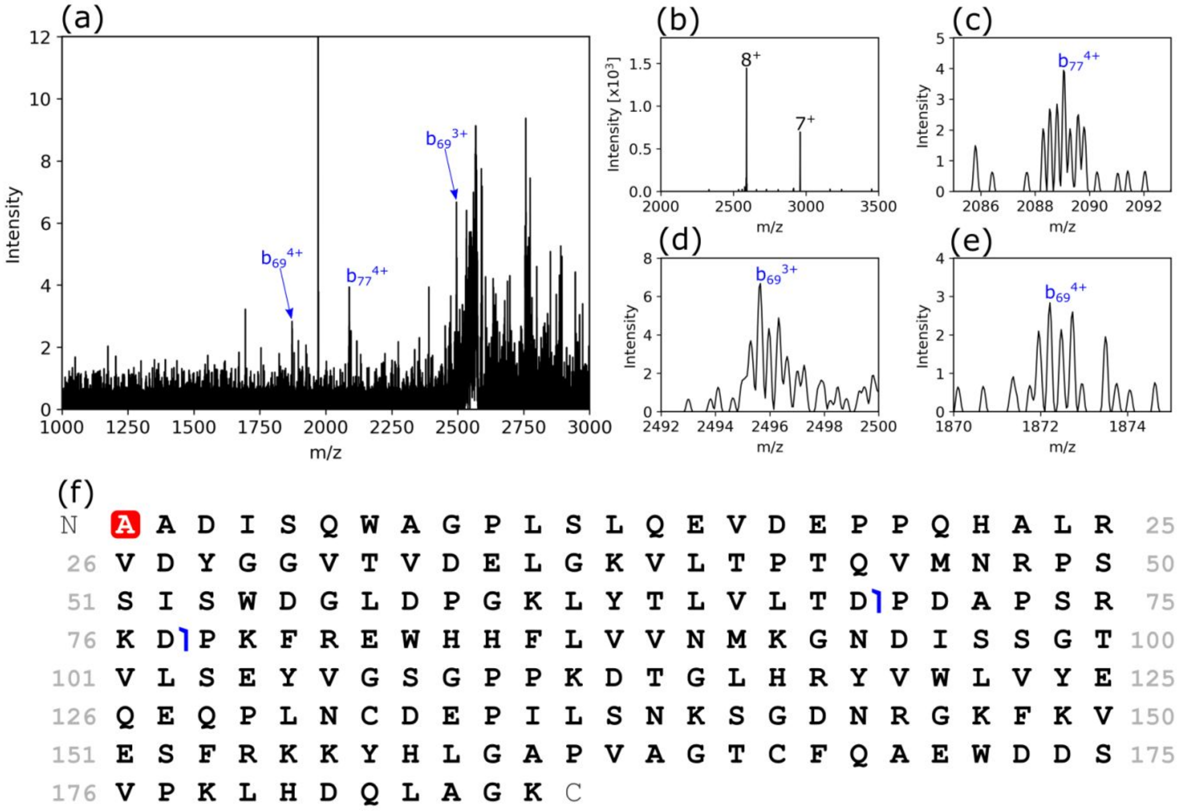

Figure S23: Identification of PEBP1. (a) CID MS^2 (NCE = 35%) mass spectrum for m/z 2589.0^{8+} ± 2.5. The charge state was determined by PTCR (b), providing the approximate MW of 20.7 kDa. PTCR also suggests that there are no other overlapping protein signals. (c-e) Zoomed CID spectra showing the isotopically resolved, but low intensity sequence ions. (f) The sequence of PEBP1. Both detected fragments are the result of Asp-Pro cleavage.

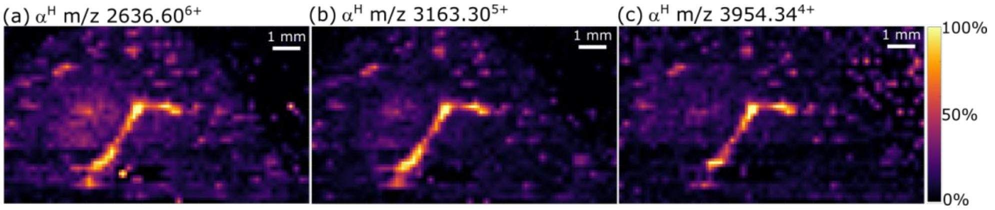

Figure S24: Ion images of three charge states of holo-alpha globin show it is predominantly localized to blood vessels.

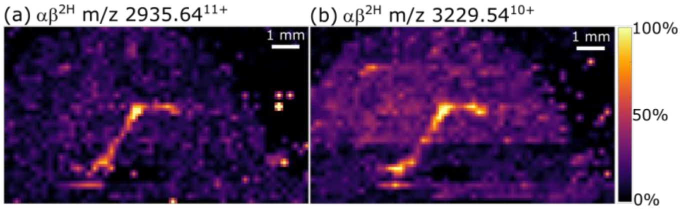

Figure S25: As with holo-alpha globin, ion images of two charge states of the $\alpha\beta^{2H}$ hemoglobin dimer show its localization in blood vessels.

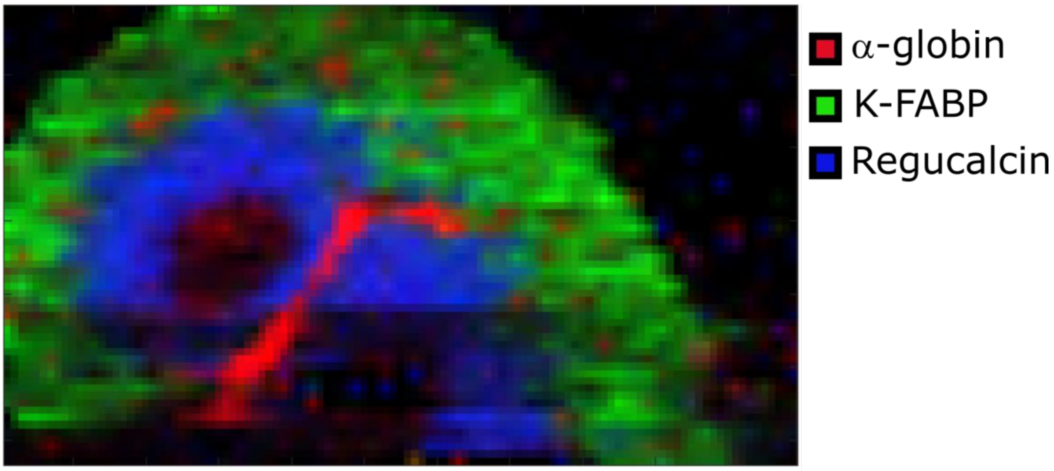

Figure S26: Composite ion image showing the presence of blood vessels (represented by α^H) amongst the bulk kidney tissue (represented by K-FABP and regucalcin).

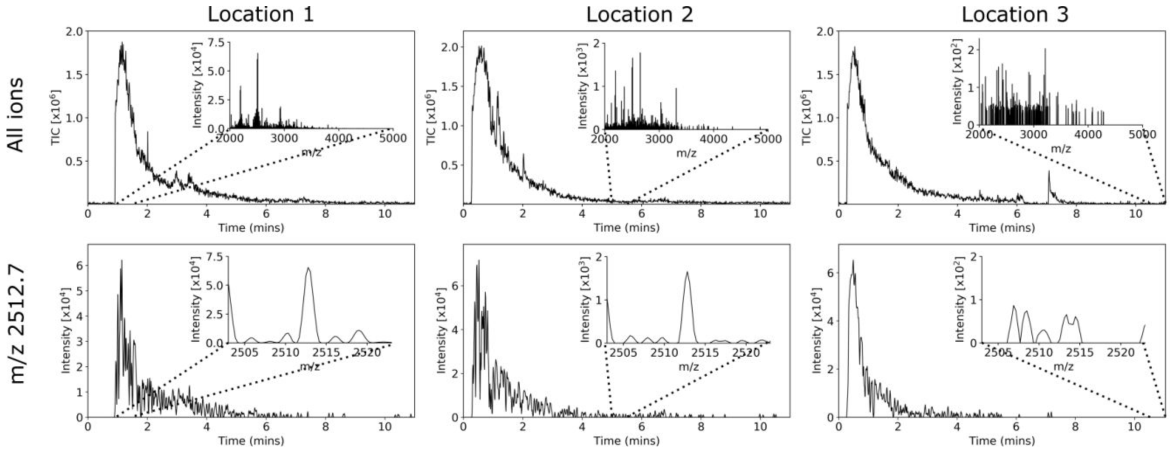

Figure S27: assessment of signal duration from three nano-DESI sample locations in rat kidney cortex tissue. Tissue was sampled without moving the probe. TIC and XIC (m/z 2512.7, K-FABP^{7+}) chronograms are shown. The TIC intensity was similar for each location. After approx. 5 mins signals decreased by over an order of magnitude in intensity compared to the first ~30 seconds, yet protein ion signals still exhibited acceptable S/N. Signals decreased to within the noise level after approx. 8 minutes. Example mass spectra at different time points are included as insets.

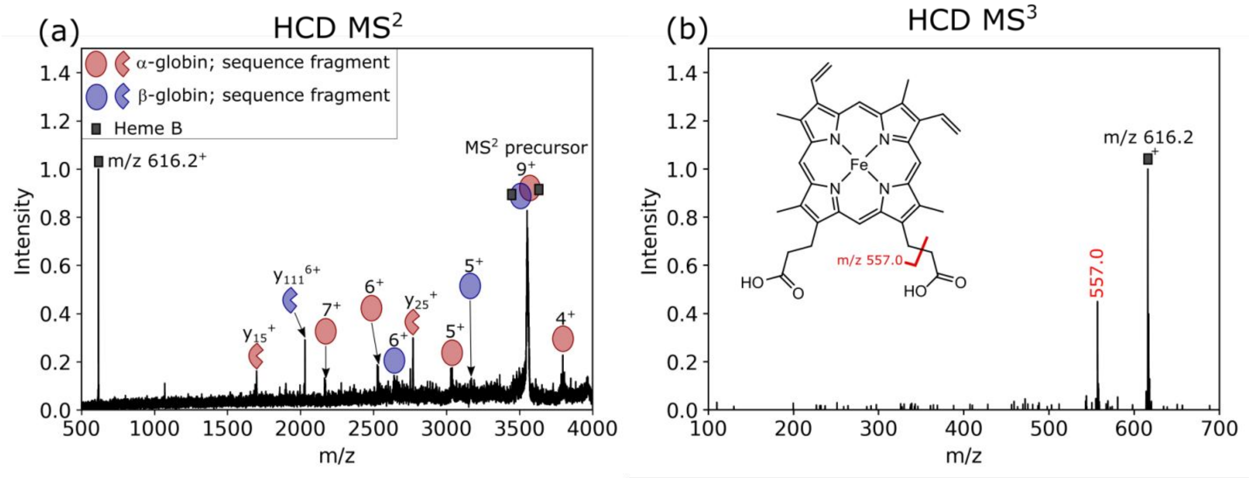

Figure S28: (a) HCD MS^2 (NCE = 32%) spectrum for $\alpha\beta^{2H}$ (m/z 3559^{9+} ± 1) sampled by native nano-DESI from a kidney blood vessel. The intact complex dissociated into apo-protein subunits (α and β) and heme B$^+$ (*m/z* 616.2) ions. Main sequence ions for the subunits were also detected. (b) HCD MS^3 (NCE^2 = 30%, NCE^3 = 62%) of heme B after dissociation from $\alpha\beta^{2H}$.

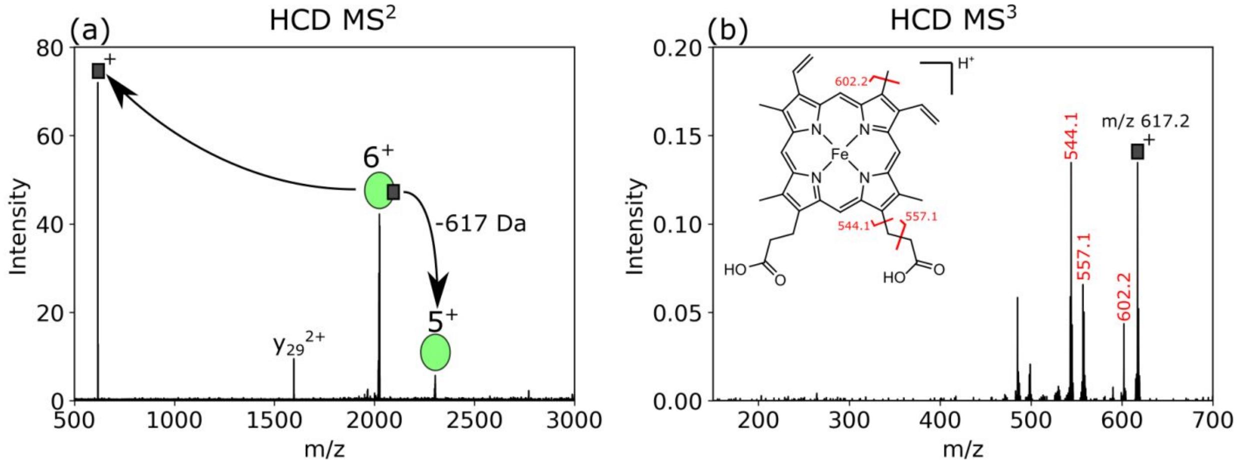

Figure S29: (a) HCD MS^2 spectrum (NCE = 35%) for cytochrome C, somatic (m/z 2022.8^{6+} ± 2) sampled by native nano-DESI from kidney cortex tissue. Predominant product ions were [heme+H]$^+$ (*m/z* 617.2)[3] and [cytochrome C - heme]$^{5+}$, indicating the cleavage of both covalent bonds between heme and cysteine residues (b) HCD MS^3 (NCE^2 = 38%. NCE^3 = 20%) of the [heme+H]$^+$ MS^2 product ion.

References

(1) Illes-Toth, E.; Cooper, H. J. Probing the Fundamentals of Native Liquid Extraction Surface Analysis Mass Spectrometry of Proteins: Can Proteins Refold during Extraction? *Anal Chem* **2019**, *91*, 12246-12254, 10.1021/acs.analchem.9b02075

(2) Wilm, M.; Mann, M. Analytical Properties of the Nanoelectrospray Ion Source *Anal Chem* **1996**, *68*, 1-8, 10.1021/ac9509519

(3) Carraway, A. D.; Burkhalter, R. S.; Timkovich, R.; Peterson, J. Characterization of heme c peptides by mass spectrometry *J Inorg Biochem* **1993**, *52*, 201-207, https://doi.org/10.1016/0162-0134(93)80041-7

(4) Hsu, C. C.; Chou, P. T.; Zare, R. N. Imaging of Proteins in Tissue Samples Using Nanospray Desorption Electrospray Ionization Mass Spectrometry *Anal Chem* **2015**, *87*, 11171-11175, 10.1021/acs.analchem.5b03389

(5) Lin, L.-E.; Chen, C.-L.; Huang, Y.-C.; Chung, H.-H.; Lin, C.-W.; Chen, K.-C.; Peng, Y.-J.; Ding, S.-T.; Wang, M.-Y.; Shen, T.-L.; Hsu, C.-C. Precision biomarker discovery powered by microscopy image fusion-assisted high spatial resolution ambient ionization mass spectrometry imaging *Anal Chim Acta* **2020**, *1100*, 75-87, https://doi.org/10.1016/j.aca.2019.11.014

(6) Chen, C.-L.; Kuo, T.-H.; Chung, H.-H.; Huang, P.; Lin, L.-E.; Hsu, C.-C. Remodeling nanoDESI Platform with Ion Mobility Spectrometry to Expand Protein Coverage in Cancerous Tissue *J Am Soc Mass Spectrom* **2021**, 10.1021/jasms.0c00354
